# Health needs of older populations affected by humanitarian crises in low- and middle-income countries: a systematic review

**DOI:** 10.1186/s13031-017-0133-x

**Published:** 2017-12-11

**Authors:** Emma Massey, James Smith, Bayard Roberts

**Affiliations:** London School of Hygiene and Tropical Medicine, 15-17 Tavistock Place, London, WC1H 9SH UK

**Keywords:** Violence, Disasters, Ageing, Health

## Abstract

**Background:**

The combination of global demographic changes and a growing number of humanitarian crises in middle-income countries that have a higher life expectancy has led to an increase in the number of older populations affected by humanitarian crises. The aim of this review was to systematically examine evidence on the health needs of older populations in humanitarian crises, including both armed conflicts and natural disasters, in low- and middle-income countries (LMICs).

**Methods:**

A systematic review methodology was used. The search strategy used terms related to older populations and humanitarian crises in LMICs. Five bibliographic databases were used, along with relevant grey literature sources. Descriptive analysis was used, and a quality assessment conducted using the Newcastle-Ottawa Scale and CASP instruments.

**Results:**

A total of 36 studies were eligible for review. The majority of the studies were cross-sectional, three were cohort studies, and four used qualitative methodologies. The main health outcomes were mental health, physical health, functioning, and nutrition. Vulnerability factors included older age, female gender, being widowed, increased exposure to traumatic events, prior mental health problems, low income and education, and rural residency. Ten studies addressed the responsiveness of health systems and access to such services. The quality of the included studies was generally low.

**Conclusions:**

There is an urgent need to strengthen the evidence base on the health needs of older populations in humanitarian crises.

**Electronic supplementary material:**

The online version of this article (10.1186/s13031-017-0133-x) contains supplementary material, which is available to authorized users.

## Background

The proportion of older people is growing faster than any other age group globally. Approximately 12% of the world’s population is aged 60 and over and the number of older people is estimated to surpass 1 billion by 2020. By 2050, there will be nearly as many people aged 60 and over as children aged under 15 [1]. Currently, two-thirds of the world’s older people live in low- and middle-income countries, which is where humanitarian crises are more likely to occur and where the humanitarian impact is greater.

An estimated 172 million people (all ages) are currently affected by armed conflict worldwide, [2] including over 59 million people forcefully displaced from their homes as either internally displaced persons (IDPs) or as refugees. Natural disasters are also estimated to affect 175 million people annually [3]. The combination of global demographic changes and a growing number of humanitarian crises in middle-income countries with higher life expectancy has led to an increase in the number of older populations affected by humanitarian crises [4–6].

Older populations are more likely to be disproportionately affected by humanitarian crises [7]. Older age is associated with increased likelihood of disability and ill health which can limit functioning and physical mobility, and impede access to health services. Ageing also increases dependency on others for financial and social support. These collective vulnerabilities put older populations at a higher risk during humanitarian crises when health risks are increased and support networks and existing social infrastructure compromised [8]. Specific health risks for older populations in humanitarian crises include: greater susceptibility to ill health, malnutrition, disability and injury; difficulties in accessing health services (including psychological services); inappropriate health services such as services not addressing non-communicable diseases which older people are more likely to suffer from; failure to collect data on health needs of older people; and broader social and economic marginalisation [9, 10].

While older populations are recognised as a vulnerable group in humanitarian crises, [11, 12] the particular needs of older populations in humanitarian crises appear poorly understood [13]. Reviews have been conducted on crisis-affected older populations, [14–16] but these have not been systematic, have focused on natural disasters only, and on high-income countries where the health needs and health sector resources and responses are likely very different compared to low- and middle-income countries (LMICs) where the vast majority of crisis-affected populations live.

The aim of this review was to systematically examine evidence on the health needs of older populations in humanitarian crises in LMICs. The specific objectives were to: identify the vulnerabilities of older populations in humanitarian crises; assess health service access and responsiveness for older populations in humanitarian crises; and evaluate the quality of the evidence.

## Methods

### Eligibility criteria

The population of interest were older populations affected by humanitarian crises in LMICs (with LMICs classified according to Word Bank listings [17]). No age limit was set as the definition of ‘older’ varies across country contexts. The study population included refugees, returnees, IDPs, and non-displaced crisis-affected people. Humanitarian crises were defined as a serious disruption of the functioning of a community or a society causing widespread human, material, economic or environmental losses which exceed the ability of the affected community or society to cope using its own resources, necessitating a request to national or international level for external assistance [18]. Humanitarian crises included both armed conflict and natural disasters [19]. Natural disaster events included earthquakes, tsunamis, floods, hurricanes, landslides, and volcanic eruptions (see Additional file 1 for the full list of events). All health outcomes were included. Research on military or veteran military populations was excluded, as were studies of an older population that had experienced a crisis at a younger age. Studies of all-age populations showing age as a risk factor but not focusing specifically on older populations were excluded.

Primary published and grey literature using quantitative and qualitative methods were included. All languages were included. No date restrictions were set (the end date was 18 July 2016).

### Search strategy

The following bibliographic databases were used: Medline, Embase, Global Health, Psychinfo, and IBSS. The search methodology consisted of three strings, with terms related to LMICs, humanitarian crises, and older populations. Free-text searching was used, and subject heading (MeSH) were also used for Medline. The search terms are listed in Additional file 1. Broad search terms such as ‘elderly’ and ‘humanitarian’ were applied to the Desastres database (mixed published and grey literature) and also to the following grey literature sources: UNHCR, MSF Field Research, HelpAge International, Handicap International, International Committee of the Red Cross (ICRC), WHO Institutional Repository for Information Sharing (IRIS), Open Grey, ReliefWeb, PsycEXTRA, ALNAP, and Google (first ten pages only).

### Study selection and data extraction

Study selection involved a four stage process: removal of duplicates (stage 1); screening by title (stage 2a) and abstract (stage 2b) and then full text (stage 2c); grey literature screening and review of the reference lists of the final selected studies (stage 3); and final review and analysis of the selected studies (stage 4).

The information extracted from the final selected studies included: author/date, location, crisis/population type, older age definition, methods, health outcomes/measurement, and findings that related to the three study objectives. Where both bivariate and multivariate analyses were performed, only multivariate results were extracted. In relation to objective one, where statistical significance tests were used, only results that were considered statistically significant (*p* < 0.05) were extracted. The study screening, data extraction, and quality assessment was conducted separately by EM and JS and any differences discussed and reconciled.

### Analysis and quality assessment

Descriptive analysis was used given the heterogeneous nature of study context, population exposure, health outcomes, and study methodologies. Findings were organised by the three study objectives, and then into commonly recurring themes. For quality appraisal, quantitative studies were appraised using the Newcastle-Ottawa Scale (NOS), [20]. with cohort studies given a score of 1–9, and cross-sectional studies given a score of 1–10 (using a modified NOS version for cross-sectional studies) [21]. For qualitative studies, the Critical Appraisal Skills Program (CASP) checklist was used, [22]. with studies given a score of 1–10. Higher scores in the quality appraisals indicate better quality. The quality appraisal process sought to identify common strengths/weaknesses, rather than to exclude studies. This review follows the PRISMA Statement for reporting systematic reviews (see Additional file 2 for the completed PRISMA checklist) [23]

## Results

### Study selection and characteristics

**Fig. 1:**
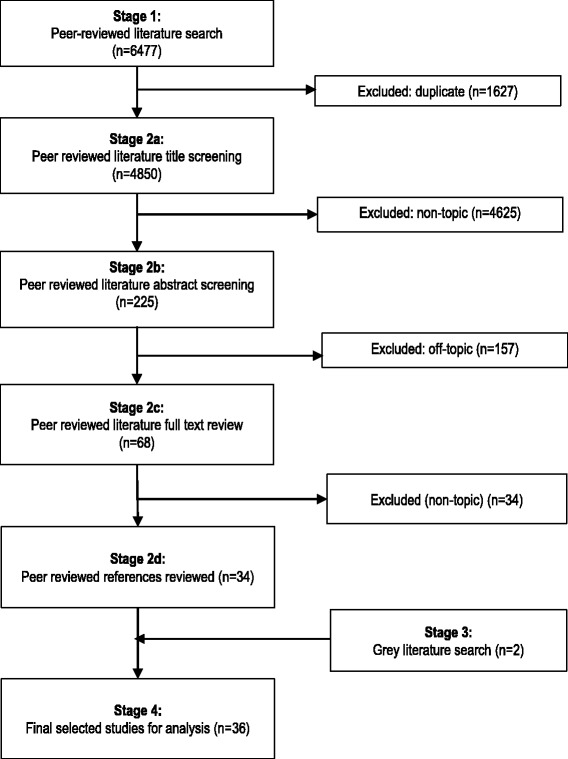
Results of screening process

Thirty-six studies met the eligibility criteria, [24–59] of which two were from the grey literature [37, 40] (Fig. 1). The most common reasons for exclusion at stage two were studies not reporting: primary research, populations in LMICs, specifically on older populations.

All 36 studies were published between 1989 and 2016, with 64% published since 2010. Two were qualitative, [25, 34] two used mixed methodologies, [40, 47] three were cohort studies, [50, 51, 55]. and the remaining 29 were cross-sectional. [24, 26–33, 35–39, 41–46, 48, 49, 52–54, 56–59]. Twenty-one studies reported on populations affected by natural disasters, [25–29, 31–34, 36, 39, 41, 42, 44, 48, 53, 54, 56–59] 14 by armed conflict, [24, 30, 35, 37, 38, 40, 43, 45–47, 49–52], and one for both crisis types [55]. The definition of older age ranged from ≥45 to ≥65 years of age, with most studies defining it as aged ≥60 years. The majority of studies were conducted in Asia (China, [29, 33, 39, 42, 54, 56–59], India, [28, 53] Sri Lanka, [34, 44] Pakistan [31, 32], and Thailand [48]), followed by the Middle East (Lebanon [30, 37, 50–52] and Iran [25–27]), sub-Saharan Africa (the Democratic Republic of Congo, [24, 43] Ethiopia, [35] Tanzania, [45–47] and Mozambique [49]), Europe (Croatia, [38] Armenia, [36] Georgia [40]), and Latin America (Honduras [41]), and one study covered 21 countries [55].

### Vulnerability factors

#### Mental health outcomes

Twenty studies reported mental health and psychosocial outcomes [25, 26, 29–31, 33, 36–42, 44, 48, 52, 53, 56, 58, 59]. A synthesis of key factors associated with mental health outcomes is presented in Table 1, with detailed results given in Table 2, and a description given below.

**Table 1:** Factors associated with better (+) and worse (−) mental and physical health outcomes

Factors	Mental health outcomes											Physical health outcomes						
	PTSD	Depression	Anxiety	Alcohol misuse	Adjustment disorder	Psych. quality of life	Psych. distress	somatic symptoms^i^	Insomnia	Bitterness/ resentment	Aggressive behaviour	Quality of Life/general health status	Physical functioning^ii^	Miscellaneous^iii^	Treatment interrupted	Mortality	Clinical complications	Malnutrition
Demographic and socio-economic:																		
Age (increasing)	- [33, 36, 39, 44, 53]	- [38, 52] [48] + [53]			-[53]	- [26, 56, 59]	- [37, 39]	- [38]		+ [38]	+ [38]	- [26, 56] - [52] β	- [27, 45, 52]			- [35, 54, 55] β	-[57]	- [24, 28, 45]
Gender (female)	- [33]	- [40, 48]	- [40, 58]			- [26, 56, 59]						- [26, 56]	-[27]			-[50]		
Single/widowed/separated (vs. married)	- [33]	-[48]				-[59]						[56]				-[51]		
Low education	- [33]	+ [52]					- [29]					- [52] β	-[27]					
Low income	- [33]	- [30, 48]				-[59]												
Loss of livelihood	- [58]																	
Loss of property																-[50]		
Low socio-economic status^iv^																		- [28]
Larger household size^v^													-[52]					
Living with others													+ [27]			-[51]		
Dissatisfaction with living conditions						- [26]						- [26]						
Living location (rural vs. urban)		- [31]				+ [26]			- [31]					-[31]	-[31]			
Low social support	- [33]	- [42]	- [52]			-[59]												
Regular religious attendance		+ [30]																
Trauma exposure & forced displacement:																		
Exposure to individual traumatic events^vi^	- [33, 36, 41, 44, 58]	- [41, 42, 48, 58]	- [58]	- [41]		- [26]	- [29, 41]					- [56]	-[27]			-[50]		
Higher intensity of exposure	- [36, 41, 58]	[42, 58]	-[58]			- [26]	- [41]									-[50]		
Forced displacement versus non-displacement		- [53]	- [53]			- [26]	- [29]									-[35]		
Longer-term displacement vs. shorter-term displacement		- [52]	+ [40] - [52]										-[52]	-[52]			-[52]	+ [49]
Injury (to respondent or family member)	-[33] [44]	-[48]	-[58]			- [26] [59]	[29]					-[26]						
Health factors:																		
Current or previous health/functioning problems	- [41]	- [30, 41]		- [41]		- [26, 56, 59]	-[29, 41]											
Illness^vii^												- [56]	-[52]			-[57]		-[24]
Poor physical functioning^ii^												- [26]						-[24]
No physical exercise																		-[24]
Malnutrition													-[46]					
History of falls																		-[24]
Needing dialysis																-[57]		

*Only quantitative studies included in Table*

*- associated with negative health outcome; + associated with positive health outcome; β did not include tests for statistical significance*

^i^ *psychosomatic measure includes individual items on symptoms of insomnia, nightmares, distraction, forgetfulness, depression, emotional numbness, and fear.* ^*ii*^ *physical functioning includes ADL, IADL, AMA and hand grip strength, and general functional status measures.* ^*Iii*^ *misc. physical symptoms include dental, visual, weight loss, eating problem, hearing, headache, and known medical problems.* ^*iv*^ *includes caste, old age pension, occupation and land ownership.* ^*v*^ *Includes living with three or more generations, living with a married child.* ^*vi*^ *Including surviving earthquake, injury during crisis event, living in high impact area, trauma exposure inventory, initial fear, bereavement.* ^*Vii*^ *includes NCD’s, sepsis, chronic illness, >3 prescription drugs/day, fever, and ARI, dementia, poor vision, difficulty walking, poor health status*

**Table 2:** Detailed results on vulnerability factors

Author, date, (reference), [quality score*]	Context (definition of older age)	Outcome of interest/study design (analysis)	Comparison group	Vulnerability factors
Mental Health Outcomes				
Ardalan et al., 2010. [25] [9/10]	Iran/earthquake (60+)	Older people’s perceptions of needs post disaster/qualitative - focus-group and interviews (content analysis).	NA (qualitative)	*Feelings of insecurity:* Not being able to protect oneself or belongings from thieves due to frail physical state and fear of being targeted for this reason. *Emotional distress:* many still very emotionally upset due to trauma during earthquake even years later. Difficult to adjust to new life after earthquake. Causes of distress: losing children, close relatives and friends, experiencing hopeless days, witnessing the destruction of historic parts of the city and losing valued documents and memorabilia.
Ardalan et al., 2011[a] [26] [7/10]	Iran/earthquake (60+)	Psychological state/quality of Life (WHOQOL-BREF)/quantitative - cross-sectional (multivariate)	Earthquake affected vs. non-earthquake affected	*Within earthquake-affected sample (regression coefficients):* higher age (−0.113, *p* = 0.003); female gender (−1.169, *p* = 0.016); urban residence (−1.043, *p* = 0.044); being unmarried (−1.144, *p* = 0.018); history of earthquake related injury (−1.542, *p* = 0.028); dissatisfaction with quality of current living place (−2.718, *p* < 0.001); functional dependence (ADL) (1.151, *p* = 0.004). *Comparison with non-earthquake-affected populations:* survivors had lower psych. State scores (mean 11.88 12.80, *p* = 0.03)
Cao et al., 2014 [29] [5/10]	China Earthquake (60+)	Psychological distress (SRQ-20/quantitative survey - cross-sectional (bivariate)	None	*Older men:* loss of family members (OR 1.32–31.53, *p* = 0.02); displacement from residence (OR 1.08–32.33, *p* = 0.04). *Older women:* higher educational level (OR 0.09–0.77 *p* = 0.02); chronic illness (OR 1.11–13.78 *p* = 0.03); loss of family members (OR 2.87–76.51 *p* = 0.00); displacement from residence (OR 3.37–37.18 *p* = 0.00).
Chaaya et al., 2007 [30] [7/10]	Palestian.refugee, Lebanon/(60+)	Depression (GDS)/quantitative - cross-sectional (multivariate)	None	Regular religious attendance (OR 0.41, *p* = 0.041); sufficient income (OR 0.42, *p* = 0.003); ADL difficulties (OR 2.05, *p* = 0.015); Illness during last year OR 2.89, p < 0.001).
Chan et al., 2009[a] [31] [4/10]	Pakistan Earthquake (45+)	Psychosocial (SRQ)/quant. Cross-sectional (bivariate)	Rural vs. urban	Rural more likely to experience feeling depressed/helpless (72% vs. 44%, *p* < 0.001). Rural more likely to experience sleeplessness (65% vs. 45%, *p* < 0.001).
Chen et al., 2012 [33] [8/10]	China Earthquake (60+)	PTSD (CAPS for DSM)/quantitative - cross-sectional (multivariate)	None	Female gender (OR 1.592 [95% CI 1.236–2.057]); aged 81 years or older (OR 1.557 [95% CI 1.057–2.292]); widowed (OR 1.464 [95% CI 1.281–1.660]); low education level (OR 1.395 [95% CI 1.073–1.804]); low monthly income (OR 1.670 [95% CI 1.401–1.992]); suffering bodily injury (OR 2.468 [95% CI 1.863–3.246]); bereavement OR 2.064 [95% CI 1.363–3.994]); low social support (OR 1.826 [95% CI 1.054–3.162].
Goenjian et al., 1994 [36] [4/10]	Armenia Earthquake (59+)	PTSD (PTSD Reaction Index) /quantitative - cross-sectional (bivariate)	Older vs. younger	*Within older population*: living in high impact zone: higher PTSD scores (*p* < 0.05) *Comparison with younger:* older people had lower PTSD score re-experiencing (2.1 vs. 2.5, *p* < 0.05); older people had higher PTSD score arousal (2.7 vs. 2.4, *p* < 0.05).
Handicap Int. & HelpAge Int, 2014 [37] [4/10]	Syrian refugees in Lebanon & Jordan/war (60+)	Psychological distress(SRQ)/ quantitative survey - cross-sectional (bivariate)	Older vs. younger	Older age populations 3 times more likely than non-elderly to show signs of psychological distress. (no *p*-value or exact figures reported)
Havelka et al., 1995 [38] [3/10]	Croatia/war (60+)	Psychosomatic Disorders (SSPD)/quantitative survey- cross-sectional (bivariate)	Older vs. younger	*Older age a risk factor for often experiencing the following outcomes related to psychosomatic feelings (older* vs. *non-elderly):* Persistent memory of stressful event: 81.5% vs. 55.2%, p 0.001; depression 80.6% vs. 52.9%, *p* = 0.001; insomnia 53.4% vs. 34.3%,*p* = 0.005; nightmares 33.0% vs. 13.8%, *p* = 0.001; distraction 32.0% vs. 14.6%, *p* = 0.001; forgetfulness: 30.1% vs. 11.3%, *p* = 0.001; emotional numbness 12.6% vs. 6.7%, *p* = 0.039; fear: 22.3% vs. 10.5%, *p* = 0.010) *Being older protective against often experiencing the following outcomes (older* vs. *non-elderly):* Bitterness and resentment towards others 18.4% vs. 36.4%, *p* = 0.001; aggressive behaviour 6.8% vs. 11.7%, *p* = 0.014.
Jia et al., 2010 [39] [8/10]	ChinaEarthquake (60+)	PTSD (PCL-C), psychiatric morbidity (GHQ-12)/quant. - cross-sectional (multivariate)	Older vs. younger	*PTSD:* older age versus non-elderly (OR 3.56, *p* = 0.002). *General psychiatric morbidity:* older age vs. non-elderly (OR 2.14, *p* = 0.005).
Johns Hopkins & Policy Studies, 2012 [40] [6/10]	IDPs in Georgia/war (60+)	Depression (GDS), anxiety (GAI)/quantitative - cross-sectional (bivariate)	Long-term(20 years) IDP vs. short-term (4 years) IDP	*Depression:* Females higher depression scores than males (*p* < 0.01) (no mean depression score reported). *Anxiety:* short-term IDP higher prevalence than long-term IDP (76% vs. 70.3%, *p* < 0.02). Females higher scores than males (*p* < 0.01) (no mean scores reported).
Kohn et al., 2005 [41] [7/10]	Hondurashurricane (60+)	Psych. distress (SRQ); alcohol misuse (SRQ); depression (DSM-IV/ICD-10); PTSD (CIDI; IES)/quantitative - cross-sectional (multivariate)	Older age vs. younger	*Risk factors for all psychopathology (except severity of PTSD avoidance subscale) in older age:* Exposure inventory (exposure to hurricane); prior “nerves” *Risk factors for PTSD and psychological distress:* living in high impact area *Older age* vs. *non-elderly:* none *(no p-values or effect measures available).*
Li et al., 2011 [42] [7/10]	China, earthquake (55+)	Depressive symptoms, stress reaction (Impact of Event Scale), sense of community (SoC Index)/quantitative – cross-sectional (multivariate)	None	High event impact, a reduced sense of community, and social support were associated (*P* < 0.05) with higher rates of depression.
Nomura et al., 2010 [44] [7/10]	Sri Lankatsunami (60+)	PTSD (IES-R)/quantitative - cross-sectional (multivariate*)	None	Increasing age by 10-year interval (coef. -0.27, *p* = 0.04) Loss of or injury to family members due to the tsunami (coef. 6.12, *p* < 0.001).
Prueksaritanond et al. 2007 [48] [2/10]	Thailand, tsunami (60+)	Depression (Zung Self-Rating Depression Scale)/Quantitative – cross-sectional /quantitative (descriptive)	None	Factors associated with increased symptoms of depression were female (odd ratio [OR] 2.81; 95% confidence interval [CI] 0.73–10.77, *p* = 0.12), aged of 65 years old and over (OR 2.0; 95% CI 0.52–7.7, *p* = 0.25), living alone such as single, divorce, or separation (OR 1.47; 95% CI 0.35–6.13, *p* = 0.44), no income was generated after the tsunami (OR 1.26; 95% CI 0.34–4.75, *p* = 0.5), hypertension (OR 1.25; 95% CI 0.34–4.59, *p* = 0.5) and loss of family members (OR 1.14; 95% CI 0.31–4.20, *p* = 0.56).
Strong et al., 2015 [52] [5/10]	Syrian & Palestinian refugees Lebanon/war (60+)	Negative emotions (SRQ)/ quantitative - cross-sectional (bivariate).	Palestinian (longer displaced) vs. Syrian refugees (shorter displaced)**	Palestinians (i.e. longer-term displaced): higher prevalence of depression (40% vs. 25%, *p* = 0.050)Palestinians (i.e. longer-term displaced): higher prevalence of feeling scared (33% vs. 18%, *p* = 0.036) *Among entire sample (Palestinian and Syrian refugees combined):* Older age (*p* = 0.017) and higher education (*p* = 0.023) with feeling depressed. Lower social support (*p* = 0.006) with anxiety.
Viswanath et al., 2012 [53] [5/10]	Indiatsunami (60+)	Psych. morbidity, adjustment disorder, PTSD, depressive episode, panic disorder, alcohol dependence, phobic disorder, anxiety (ICD-10 criteria)/ quantitative - cross-sectional (bivariate)	Older age vs. youngerDisplaced vs. non-displaced	*Total sample:* (older age vs. non-elderly): Older people more likely to suffer from adjustment disorder (50% vs. 37%, *p* = 0.030).*Displaced (older age* vs. *non-elderly):* Older people less likely to suffer from depressive episodes (6% vs. 20%, *p* = 0.019). *Non-displaced:* (older age vs. non-elderly): Older people less likely to suffer depressive episode (7% vs. 27%, *p* = 0.002). Elderly more likely to suffer PTSD (18% vs. 8%, *p* = 0.036). *Within older age sample (older age* vs. *non-displaced):* non-displaced more likely to suffer adjustment disorder (61% vs. 17%, *p* = 0.001). Displaced more likely to suffer depressive episode (44% vs. 7%, *p* < 0.001) and unspecified anxiety disorder (22% vs. 4%, *p* < 0.011).
Wu et al., 2015 [56] [5/10]	China, flooding (60+)	‘Health related quality of life’ (HRQoL), incl. Role limitations due to emotional problems, mental health/ quantitative – cross-sectional (multivariate)	Pre-flood rural older people (from National Health Services Survey 2008)	Self-reported HRQoL lower in those aged 80–99 (vs. 60–79), lower in those who are single (vs. married), lower in those with poor sleep patterns, lower in those with pre-existing chronic diseases, lower if hospitalised within the last year, lower if living alone (vs. with spouse),
Zhang et al., 2012[b] [58] [9/10]	China earthquake (60+)	PTSD (PCL-C); anxiety/dep. (HSCL-25)/ quantitative - cross-sectional (multivariate).	None	*PTSD:* loss of livelihood (OR 3.06 [95% CI 1.30–7.21]); initial fear (OR 1.74 [95% CI 1.16–2.54]). *Anxiety:* female (OR 2.03 [95% CI 1.09–3.39]); bereavement (OR 2.59 [95% CI 1.17–5.77]); injury (OR 2.03 [95% CI 1.03–4.11]). *Depression:* Initial fear (OR 1.44 [95% CI 1.03–2.01]).
Zhang et al., 2012(c) (5/10] [59]	China earthquake (60+)	Quality of Life (QoL) score/quantitative – cross-sectional (bivariate)	National average	Lower QoL scores are associated (*p* < 0.5) with: female gender; age over 70; single; lower income; non-smoker; disability in self or family member, poor family relationship
Physical health outcomes				
Andre et al., 2013 [24] [3/10]	Rural Democratic Republic of Congo/war (65+)	Nutritional status (*MNA-SF/LF)/*quantitative - cross-sectional (bivariate)	None	Differences in nutritional status (normal vs. malnourished): mean age (years) 68.4 (+ − 4.0) vs. 74(+ − 6.7) (*p* < 0.001); BMI <18.5 15.7% vs. 81% (*p* < 0.001); smoking 31.4% vs. 2.9% (*p* < 0.001); physical exercise (1–5/week) 100% vs. 2.9% (*p* < 0.001); >3 prescription drugs/day 19.6% vs. 68.6 (p < 0.001); ADL limitation 50.9% vs. 87.6% (*P* < 0.001); IADL limitation 11.8% vs. 94.3% (P < 0.001); history of falls: 35.8% vs. 61% (*p* = 0.003)
Ardalan et al. 2011[a] [26] [7/10]	Iran earthquake (60+)	Physical Quality of life (QoL) (*WHOQOL-BREF)/* quantitative - cross-sectional (multivariate)	Earthquake affected vs. non-earthquake affected	*Within earthquake affected sample (regression coefficients):* Higher age (−0.113) (*p* = 0.001); being female (−1.320) (*p* = 0.017)Being injured due to earthquake (−2.370) (*p* = 0.006); dissatisfaction with quality of current living place (−2.411) (p < 0.001); functional (ADL) dependence (−1.963) (*p* = 0.001)
Ardalen et al., 2011[b] [27] [7/10]	Iran earthquake (60+)	Functioning (ADL and IADL, 2 months, 2 years and 5 years after event)/quantitative - cross-sectional (multivariate)	None	*Determinants of functional capacity (regression coefficients, p < 0.05):* Model 1 (controlled for all eligible factors *except* ADL and IADL scores at preceding time period):1. ADL scores at 2 months after the earthquake: age (−0.60); living with others (−.68); and chronic diseases (−0.66). 2. ADL scores at 5 years after the earthquake: age (−0.64); gender (0.41); living with others (−0.86); and chronic diseases (−0.40) 3. IADL scores at 2 months and 5 years after the earthquake: age (−1.64 & -1.61); education (1.44 and −1.47); study area (1.21 and 1.12); living with others (−1.78 & -1.91). Model 2 (controlled for all eligible factors *including* ADL and IADL scores at preceding time period): 1.ADL score at two months after the earthquake: age (−0.42), living with others (−0.49), and ADL before the earthquake (0.81). 2. ADL score at 5 years after earthquake: gender (0.16), living with others (0.12), and ADL at 2 months after earthquake (1.03). 3. IADL score at 2 months after earthquake: age (−0.85); living with others (−0.80); and IADL before the earthquake (0.68). 4. IADL score at 5 years after earthquake: IADL at 2 months after the earthquake (0.99).
Arlappa et al., 2009 [28] [4/10]	Rural India/drought (60+)	Chronic Energy Deficiency (CED) and BMI/quantitative – cross-sectional (descriptive)	None	*Age (70+* vs. *60–69):* higher CED in both genders (males: 59.2% vs. 47.5%; females: 56.6% vs. 45.8%, *P* < 0.001). *Age (18–59* vs.*60+):* higher CED among older adults (males: 51.8% vs.38.1%; females: 48.5% vs. 40.5%,,*p* < 0.001). *Socio-economic factors:* Caste (scheduled caste and scheduled tribe 57% vs. Backward caste and others: 44.2%, (p < 0.001); pension (availing: 55.1%, not availing: 49.7% and not required: 45.8%, *p* < 0.05); occupation (non-agricultural: 53.4%, Agricultural: 52.1% and others 46.1%, (*p* < 0.001); total land acres (none: 49.3%, 0.01–2.5: 53.7%, 2.5–5: 52.3% and >5: 43.1%, *p* < 0.001).
Chan et al., 2009[a] [31] [4/10]	Pakistan earthquake (45+)	Dental, visual, eating, hearing, headaches,dizziness,muscle/ joint pain (all SRQ); health seeking behaviour/health access/quantitative - cross-sectional (bivariate).	Rural vs. urban	*Rural prevalence (%) compared to older people in urban areas*: β Dental (100 vs. 25) (*p* < 0.0001); visual (75 vs. 38) (p < 0.0001); weight loss (75 vs. 50, p = 0.001); eating problem (87 vs. 50) (*p* = 0.002); hearing (54 vs. 40) (*p* = 0.043); headache (40 vs. 23, p = 0.043); having known medical problem for which never having had treatment (65 vs. 30, p < 0.001); having known medical problem with treatment discontinued (80 vs. 40, *p* < 0.001) *Urban – higher prevalence (%) compared to older age in rural areas:* β a known underlying medical problem (38 vs. 25, *p* = 0.02).
Godfrey & Kalache, 1989, [35] [3/10]	Ethiopian refugees in Sudan/war and famine (45+)	Mortality rates; prevalence of disability (SRQ)/quantitative - cross-sectional (descriptive)	None	*Age-specific mortality rates estimated since arriving in Sudan 1 year (using population estimate as denominator):* 45–49 years: 5/1000 per year (*N* = 1); 50–59 years: 35/1000 per year (*N* = 5); 60+ years: 273/1000 per year (*N* = 3). Age-specific mortality rates for 2-year period prior to migration (denominator all those reported in Tigray households during this period): 45–49 years: 14/1000 per year (N = 5); 50–59 years: 41/1000 per year (*N* = 4); 60+ years: 91/1000 per year (*N* = 8).
Pieterse et al., 1998 [45] [6/10]	Rwandan refugees in Tanzania/war (60+)	BMI, AMA, AFA, MUAC/quantitative - cross-sectional (bivariate)	Older vs. younger	*Older higher prevalence of malnutrition (BMI < 18.5):* Men (23.2% vs. 15.0%, *p* < 0.05); women (15.1% vs. 10.9%, *p* < 0.05) *Older lower mean AMA (important in relation to ability to remain active and independent):* Men 50–59, 60–69 and 70+ (34.7, 32.3, 30.9, respectively, p < 0.05); women 50–59, 60–69 and 70+ (35.1, 33.0, 31.5, respectively, *P* < 0.05).
Pieterse et al., 2002 [46] [8/10]	Rwandan refugees in Tanzania/ war (50+)	Handgrip strength/quantitative - cross-sectional (multivariate)	Older vs. younger	*Men:* BMI contributes 5.7% to variation in Handgrip strength. (coef 0.262, *p* < 0.001); AMA contributes 10.2% to variation in handgrip strength (coef 0.303, *p* < 0.001).Women: BMI contributes 3.5% to variation in handgrip strength (coef 0.188, *p* < 0.001); AMA contributes 2.8% to variation in handgrip strength (coef 0.153, *p* < 0.001).
Pieterse & Ismail, 2003 [47] [4/10]	Rwandan refugees in Tanzania/war (50+)	Perceptions of nutritional risk factors by older persons/ qualitative interviews (ranking methodology)	None (qualitative)	*Older people’s perceptions of main problems of the less well-off were:* physical impairment; no purchasing power, income, tools and utensils; no people to provide assistance and moral support, social isolation. *Older people’s perceptions of who were the most vulnerable: w*idows and widowers; physically impaired and disabled; those living alone, have no children living nearby, have care-giving responsibilities (for example for young children or old spouse).
Ramji &Thoner, 1991 [49] [2/10]	Displaced in Moz- mbique/Zimbab-we/war (45+)	BMI/quantitative - cross-sectional (descriptive)	None	Older women in Mozambique (displaced on average 6 months) had a mean BMI significantly lower than older Mozambique women displaced to Zimbabwe (displaced on average 2 years). BMI 17.3 vs. 21.1 (*p* < 0.001)
Sibai et al., 2001 [50] [9/9]	Lebanon, war (50+)	Mortality/quantitative – cohort (multivariate)	Participants in a 1983 community-based health survey	Women exposed to human losses had a significant excess risk of both CVD and total mortality (RR 3.37 and RR 2.28 respectively). Exposure to property losses carried a greater mortality risk for men. Positive trend in the rate ratios for mortality endpoints with an increase in the intensity of exposure to a cumulative number of war events.
Sibai et al., 2007 [51] [8/9]	Lebanon/war (50+)	All-cause mortality, cardiovascular mortality (ICD-9)/ quantitative - cohort (multivariate)	None	*Cardiovascular mortality:* Men: unmarried (RR 2.50 [95% CI 1.28–4.89]); living with ≥3 generations (RR 1.99 [95% CI 1.32–3.00]); living with married child (RR 1.63 [95% CI 1.03–2.57]). Women: none *All-cause mortality:* Men: widowed/divorced/separated (RR 1.63 [95% CI 1.06–2.52]); living with ≥3 generations (RR 1.56 [95% CI 1.12–2.15]); living with married child (RR 1.70 [95%CI 1.19–2.43]).Women: living with married child (RR1.55[95%CI1.04–2.32]).
Strong et al., 2015 [52] [5/10]	Syrian & Palestinian refugees inLebanon/war (60+)	Negative emotions (using SRQ), and functional status (Katz Index of Independence in Activities of Daily Living/ quantitative - cross-sectional (bivariate).	Palestinian (i.e. longer-term displaced) vs. Syrian refugees (i.e. shorter-term displaced)**	Palestinians (i.e. longer-term displaced) higher prevalence of: hypertension (86% vs. 53%, *p* < 0.001); diabetes (81% vs. 38%, *p* < 0.001); eye disease (28% vs.16%,p 0.002); lung disease (44% vs.11%,p < 0.001); digestive tract disease (23% vs. 9%, p0.010); difficulty walking (65% vs. 39%,p 0.002); impaired vision (70% vs. 13%, *p* < 0.001); impaired hearing (49% vs. 8%, *p* < 0.001) Palestinians (i.e. longer-term displaced) lower prevalence of Arthritis, injury or back pain (7% vs. 31% *p* 0.007); *Among entire sample (Palestinian and Syrian refugees combined):* Older age (*p* = 0.002) and larger household size (*p* = 0.003) with worse functional status. Older age and lower educational status with worse self0reported health status. β
Wen et al., 2010 [54] [7/10]	China, earthquake (65+)	Mortality, physical injury/quantitative – cross-sectional (descriptive)	All earthquake-affected patients	Extremities the most common location of trauma in older patient admitted to hospital. Mortality significantly higher in this age group - secondary to e.g. thoracic visceral and craniocerebral injuries. Admission of older age patients peaking on the third, fifth, and eighth days. β
Wong et al., 2015 [55] [5/9]	21 crisis-affected countries (conflict & natural disasters)	Intra-operative mortality & surgical procedure types – retrospective cohort of routine data from 93,385 operative cases (11% were older people) at MSF facilities, June 2008 to Dec 2012 (descriptive)	Younger populations (<50) from same crisis-affected populations	Intra-operative mortality increased with each age stratum from 60 years onwards
Wu et al., 2015 [56] [5/10]	China, flooding (60+)	HRQoL: physical functioning, role limitations due to physical illness, bodily pain, general health perceptions, vitality, social functioning/quantitative – cross-sectional (multivariate).	Pre-flood rural older age (National Health Services Survey 2008)	Self-reported physical health lower in those aged 80–99 (vs. 60–79), lower in those who are single (vs. married), lower in those with poor sleep patterns, lower in those with pre-existing chronic diseases, lower if hospitalised within the last year, lower if living alone (vs. with spouse), lower in those with illness in the last two weeks, lower in females
Zhang et al., 2012[a] [57] [7/10]	China earthquake (65+)	Clinical features and outcomes of crush patients with acute kidney injury, mortality rate/ quantitative - cross-sectional using medical records (multivariate)	Older age vs. younger	*Clinical and lab findings: (older age* vs. *younger):* higher systolic pressure (131.9 vs. 115.2, *p* = 0.001); lower incidence of oliguria (13.2% vs. 41.0%, *p* = 0.001) ; lower creatinine (220.4 vs. 352.6, *p* = 0.001); lower potassium (4.1 vs. 5.3, (p < 0.001); lower serum phosphorus (1.2 vs. 1.9, (p < 0.001); lower creatinine (8173.6 vs. 57,423.0, *p* = 0.001). *Trauma events and medical complications (older age* vs. *younger): l*ower % of extremity crush injury (71.1% vs. 88.6%, *p* = 0.004); higher proportion of thoracic trauma (35.6% vs. 18.7%, *p* = 0.016); higher proportion of extremity fracture (42.2% vs. 19.9%, p = 0.002); higher proportion of rib fractures (26.7% vs. 7.2%, p = 0.002); higher proportion of vertebral fractures (17.8% vs. 6.7%, *p* = 0.020); higher proportion of pneumonia (42.2% vs. 25.9%, *p* = 0.035) *Risk factors for death:* older people receiving dialysis had higher mortality rate compared to younger adults (62.5% vs. 10.5%, *p* < 0.001). *Risk factors for death in older people (controlling for BP, no of injuries, ISS, thoracic trauma, ARDS, sepsis/or dialysis): d*ialysis (OR 15.14, *p* = 0.011); sepsis (OR 13.24, *p* = 0.030).

*For studies using both bivariate and multivariate analysis only multivariate factors were extracted. Only significant associations were extracted (p < 0.05) for studies that conducted statistical tests*

β Did not include tests for statistical significance

*For detailed results on quality assessment, please email corresponding author

**Length of displacement is an assumption by review authors based on history of Palestinian and Syrian displacement and not explicitly reported by study authors (Strong et al.*, 2015)*

##### Demographic and socio-economic factors

Twelve studies observed associations between older age and post-traumatic stress disorder (PTSD), [33, 36, 39, 44, 53], depression, [38, 48, 52]. worse psychological quality of life, [26, 56, 59] psychological distress, [37, 39] symptoms suggestive of psychosomatic disorders, [38] and adjustment disorder [53]. However, one study in the Andaman and Nicobar Islands, India, following the 2004 tsunami, reported that older age was a protective factor against major depressive episodes [53].

Five studies observed that female gender was associated with PTSD, [33] depression, [40, 48] worse psychological quality of life, [26, 56, 59] and anxiety [40, 58]. Low education was associated with PTSD [33] and psychological distress [29], but was protective against depression among refugees in Lebanon [52]. Low income was associated with PTSD, [33] depression, [30, 48] and quality of life, [59]. while loss of livelihood was associated with PTSD, [58] all among earthquake survivors in China.

Being widowed, unmarried, single or separated were associated with PTSD, [33] depression, [48] and worse psychological quality of life [26, 56, 59]. Reduced social support was associated with PTSD, [33] depressive symptoms, [42] quality of life, [59] (all in China) and anxiety (refugees in Lebanon), [52] as was a reduced sense of community with depressive symptoms (China) [42]. Regular religious attendance was associated with reduced probability of depression among refugees in Lebanon [30].

One study following the 2003 Bam earthquake in Iran observed that rural residents scored a higher psychological quality of life than affected urban residents [26]. However, rural residents were more likely than urban residents to report sleeplessness and a feeling of depression or helplessness after the 2005 Kashmir earthquake in Pakistan [31].

##### Exposure to crises, traumatic events and forced displacement

Five studies observed greater intensity of exposure to crises increased the risk of PTSD, [36, 41, 58] depression, [42, 58] anxiety, [58] worse psychological quality of life, [26] and psychological distress [41]. Three studies showed an association between bodily injury from a crisis exposure (most commonly in the context of a natural disaster) with PTSD, [33] anxiety [58] and worse psychological quality of life [26]. Three studies with earthquake and Tsunami survivors in Sri Lanka, Thailand, and China reported the effects of loss, disability or injury of a family member on PTSD, [44]. depression, [48] psychological distress, [29] and quality of life [59].

Three studies assessed PTSD levels at 1 year, [58] 15 months, [39] and 3 years [33] after the Wenchuan earthquake in China, and observed PTSD remained high many months and years after the earthquake. The study in Lebanon of long-term Palestinian refugees and shorter-term Syrian refugees found that Palestinian refugees had higher levels of depression and experiencing fear than Syrian refugees (the time period of displacement was not recorded in the study but we have assumed that Palestinian refugees had been displaced for a longer time than Syrian refugees given their histories of forced displacement) [52]. A qualitative study in Iran found that older populations experienced a significant amount of emotional distress years after the Bam Earthquake, and they found it difficult to move on from the earlier crisis events [25]. Conversely, a study in Georgia found that IDPs displaced for a shorter period of time were more susceptible to depression [40].

Forced displacement and dissatisfaction with current living conditions after a crisis was related to worse psychological quality of life [26] and psychological distress [29] among earthquake survivors in Iran and China respectively, [26, 29] and anxiety disorder among Tsunami survivors in India [53]. However, the 2004 Andaman and Nicobar study reported that remaining in the crisis-affected area increased the likelihood of suffering from adjustment disorder [53].

##### Health problems and illness

Five studies found that current or prior health conditions including chronic conditions, ‘prior nerves’, physical mobility constraints and limited functioning increased the likelihood of PTSD, [41] depression, [30, 41] alcohol disorder, [41] poor psychological quality of life, [26, 56, 59] and psychological distress [29].

#### Physical health, functioning and nutritional outcomes

Ten studies reported on various physical health outcomes in older populations, [26, 27, 31, 35, 50, 51, 54–57] and six studies reported on nutritional outcomes [24, 28, 45–47, 49] (although three of these were from the same larger study [45–47]). These results are synthesised in Table 1, with details given in Table 2, and described below.

##### Demographic and socio-economic factors

Older age was associated with lower physical quality of life among earthquake and flood survivors in Iran and China, [26, 56] lower physical functioning in Iran, Rwanda, and Syrian refugees in Lebanon, [27, 45, 52] higher mortality risk among Ethiopian refugees in Sudan and earthquake survivors in China, [35, 54] worse nutritional outcomes, [24, 28, 45] worse clinical outcomes (except for oliguria) among patients with traumatic injuries following the 2008 Sichuan Earthquake in China, [57] and higher intra-operative mortality in 21 countries [55].

Female gender was associated with lower physical quality of life [26] and physical functioning among earthquake survivors in Iran, [27] and with cardiovascular and all-cause mortality among war-affected persons in Lebanon [50] Low education was associated with worse physical functioning among earthquake survivors in Iran [27] and self-reported health status among refugees in Lebanon [52]. Lower socio-economic status was associated with a higher prevalence of chronic energy deficiency following a period of severe drought in India [28]. The loss of property had a greater mortality risk for war-affected men in Lebanon [50].

Being single, divorced, widowed or separated increased the risk of death from cardiovascular disease and all-cause mortality in Lebanon [51] and worse self-reported health among flood survivors in China [56]. A qualitative study with Rwandan refugees in Tanzania found that older populations perceived that those who lived alone and had no family or spouse to care for them were at the greatest risk of poor nutrition, citing reduced income and inadequate support networks [47]. Conversely, living with others was associated with a worse physical functioning score in the older Bam earthquake survivors in Iran [27]. The study of Palestinian refugees in Lebanon observed living in a larger household size was associated with worse functional status [52].

A study of survivors of the 2005 Kashmir earthquake in Pakistan observed a higher prevalence in rural areas than urban areas of dental, visual, eating, and hearing problems, headache, dizziness, muscle and joint pains, and of established yet untreated medical problems [31].

##### Trauma exposure and forced displacement

The study from Lebanon observed excess risk of both cardiovascular and total mortality following human loss (deaths of close relatives/friends, injuries, kidnappings, and serious threats) among women, and of cumulative exposure to war events among men and women [50]. Flood-affected populations in China reported lower health related quality of life compared to non-flood affected populations [56]. The study of Ethiopian refugees in Sudan observed mortality rates markedly increased one year after migration compared to the pre-migration period [35]. The study of displaced Palestinian and Syrian refugees in Lebanon observed longer-term Palestinian refugees were more likely to suffer from NCD’s, poor physical functioning, physical limitations and impaired vision and hearing when compared to shorter-term Syrian refugees [52]. However, a study of Mozambican refugees reported lower poorer nutritional status among those who had been displaced for a shorter period of time [49].

##### Health-related factors

One study in the Democratic Republic of Congo observed that taking no physical exercise, taking multiple prescription drugs, and limited mobility and functioning were associated with malnutrition [24]. The study with Rwandan refugees in Tanzania reported that malnutrition had a negative effect on physical functioning in terms of handgrip strength [46]. The related qualitative study reported perceptions that older people who were physically impaired were at greater risk of poor nutrition due to reduced income [47]. The study of flood survivors in China reported poor sleep patterns, diagnosed chronic disease, and hospitalisation in the preceding year were all associated with poor physical health [56]. The study of refugees in Lebanon noted dementia, poor vision, difficulty walking, poor self-reported health status were associated with lower functional status [52].

### Health service access and responsiveness

Ten studies examined aspects of health service access and responsiveness for older populations (Table 3) [25, 27, 31, 32, 34, 39, 40, 43, 52, 56]. The majority of these studies were based on descriptive self-reporting, with no statistical tests.

**Table 3:** Health Service Needs, Utilisation and Responsiveness

Author, date, reference, (quality score) ^b^	Context	Definition of older age	Outcome of interest/ study design (analysis)	Health service needs/utilization	Responsiveness of services
Ardalan et al., 2010. [25] (9/10)	Iran earthquake	60+	Perceptions of older persons of post-disaster needs. Qualitative - focus-group and interviews (content analysis)		*Inappropriate services:* Aid-agencies distributing food and equipment without attentions to special needs of older people. Not receiving appropriate attention for their physical limitations. Unable to benefit from some provided supplies due to physical constraints. Affronts to dignity in the ways that the relief aid was provided. Aid delivered in a competitive way that disadvantaged older people. Not consulted about needs. Due to physical restrictions, older people would have preferred assistance to be given in their homes. Perception that asking for help in public was degrading. Maintaining respect was a priority.
Ardalen et al., 2011 (b) [27] (7/10)^a^	Iran earthquake	60+	Accessing medical services & difficulty measure (SRQ)/ cross-sectional (multivariate)	Difficulty accessing medical services 2 months after event: 58.6% (not significantly associated with ADL or IADL score)	
Chan et al., 2009(a) [31] (4/10)	Pakistan earthquake	45+	Attendance and conditions treated at clinic/clinic records review (bivariate)	*Utilisation*: fewer older people utilized rural clinic (compared to urban): 14% vs.26% (no significance test available) suggesting geographical barrier. *Gender:* Men predominant users of rural clinic 70% (vs. females 30%). Women’s use of services were inversely related to travel distance to the clinic. When only male doctors available (in rural clinic) attendance decreased by 30%. Men had no access to psychosocial support (available programs targeted only women and children).	Absence of documentation regarding chronic diseases in clinic records. Only acute medical problems were treated despite findings of existence of chronic conditions in 38% of rural study sample (physical examination).
Chan, 2009(b) [32] (4/10)	Pakistan earthquake	Not specified	Healthcare provider perspective on health needs of older people/quantitative - stakeholder survey (descriptive analysis)		*Planning:* 6.6% had planning/consideration of older poeple in their available programs. No local organizations had awareness of guidelines for geriatric services.40% of INGO’s had heard of guidelines but none had planned for such services.1 national agency had guidelines and had considerations in their program. *Awareness:* No one provided geriatric specific services in the emergency programs. *Capacity:* No one had staff trained in geriatric sub-specialities. 40% had staff trained in NCD management. 60% had drugs to treat common geriatric illnesses. 20% had access to mobility aids. INGO’s performed the worst in terms of age- and gender sensitive programs. 80% of INGO’s had relevant drugs. No INGO provided geriatric specific services or mobility aids.
Duggan et al., 2010 [34] (7.5/10)	Sri Lanka tsunami	60+	Perceptions of older people on disaster response and preparedness/qualitative (content analysis)	Problems accessing government services (scarcity of services and cost of transport); older people excluded from rehabilitation programs due to targeting; lack of outreach programs; strong feelings of self-responsibility and inability to affect the situation.	*Inclusion and consultation:* Agencies failing to consult older people on needs. Older people reported no exposure to disaster preparedness information. Perception of unfair system of distribution of aid. Lack of relief addressing long-term needs. Rarely consulted on their needs for accessing services.
Jia et al., 2010 [39] (8/10)^a^	China earthquake	60+	PTSD (PCL-C), general psychiatric morbidity (GHQ-12)/quantitative - cross-sectional (multivariate)	Younger adults: more utilization of mental health services (19.6 vs.12.3%). Difference non-significant (*p* = 0.10).	
Johns Hopkins & Institute for Policy Studies, 2012 [40] (8/10)	IDPs in Georgia/war	60+	Older people’s perspective on current problems for internally displaced older adults/qualitative (thematic analysis)	*Health access problems:* money for medications, no or insufficient health insurance, expensive and ineffective medical treatments.	
Lutala et al., 2010 [43] (3/10)	Democratic Republic of Congo /war	60+	Healthcare seeking behaviours of older people (SRQ) /quantitative - cross-sectional (descriptive)	*Knowledge*: Knowledge of modern health structure 37.2%; unaware of any health facility 6.4%; unsure of how to answer the question 57% *Health utilisation:* private facility and traditional healer: 56.6%; public health facility: 3.3%; facility preference - public health facility 36%, private health facility: 1.2%	
Strong et al., 2015 [52] (5/10)	Syrian & Palestinian refugees in Lebanon/war	60+	Reasons for not seeking care/quantitative - cross-sectional (descriptive)	98.5% reported difficulties in accessing health care. Main reasons for not seeking care: financial 79%; lack of knowledge of where to go 12%; physical inability to travel 4%. 97% reported difficulties in accessing medicines. Main reasons for not accessing medicines: financial 87%; lack of knowledge of where to go 7%; physical inability to travel 3%.	
Wong et al., 2015 [55] (5/9)	21 crisis-affected countries (conflict & natural disasters)	(50+)	Intra-operative mortality & surgical procedure types – retrospective cohort of routine data (93,385 operative cases, 11% older people) at MSF facilities, June 2008 to Dec 2012 (descriptive)	A lower proportion of urgent surgical cases when compared to younger age groups (<50 years). Most commonly performed surgical procedures for older people included herniorrhaphies, simple and extensive wound debridement, abscess incision and drainages, minor tumorectomies, and urological procedures	
Wu et al., 2015 [56] (5/10)^a^	China, flooding	60+	Healthcare seeking behaviour	Utilisation: two-week health-care seeking rate was significantly higher in the post-flood group (*p* = 0.013) (vs. reference population)	

^a^ *These were the only studies that conducted tests of statistical significance*

^b^ *For detailed results on quality assessment, please email corresponding author*

Five studies reported how older populations had difficulty accessing medical services [27, 31, 34, 40, 52]. Reasons included: a lack of financial resources for treatment and transport; [34, 40, 52] the systematic exclusion of older populations from programmes targeting other groups; [31, 34] limited knowledge about appropriate facilities; [52] an absence of outreach programmes; [34] and inability to travel to clinics [52]. The study of Syrian and Palestinian refugees in Lebanon reported over 97% of older populations experienced difficulties accessing medical services and medicines [52].

Four studies assessed health service utilisation [31, 39, 43, 56]. Rural residents in post-earthquake Kashmir were less likely to utilise health services than urban residents, particularly women – with clinician gender playing an important role [31]. A study in eastern Democratic Republic of Congo found a very small proportion (3.3%) of older populations utilised health services when they were sick [43]. A study of flood-affected residents in Bazhong in China found the two-week healthcare-seeking rate was significantly higher than non-flood affected older populations in Sichuan province [56]. The study of surgical outcomes in 21 countries observed a lower proportion of urgent surgical cases when compared to younger age groups (<50 years); and the most commonly performed surgical procedures for older people included herniorrhaphies, simple and extensive wound debridement, abscess incision and drainages, minor tumorectomies, and urological procedures [55].

Four studies addressed the responsiveness of health services to the needs of older populations [25, 31, 32, 34]. The qualitative study of Bam earthquake survivors in Iran revealed they perceived services to be inappropriate, with a lack of respect paid to the needs and dignity of older people [25]. Another qualitative study of survivors of the 2004 Tsunami in Sri Lanka observed that older populations felt that they were not adequately consulted about their specific needs [34]. The two remaining studies assessed service responsiveness from the perspective of health service providers after the 2005 Kashmir earthquake in Pakistan, and found that many of their medical problems were undertreated, [31] and the level of awareness of the special needs of older populations was inadequate among all types of healthcare providers [32].

### Quality of the evidence

A commonly recurring issue with the quantitative studies was the limited statistical analysis, including a substantial proportion of the studies only performed descriptive bivariate analysis and so could not control for potential confounding [24, 28, 29, 31, 35–38, 40, 42, 45, 48, 49, 52–56]. Only four studies reported descriptive prevalence without calculating confidence intervals or statistical significance tests where it would have been appropriate [31, 32, 35, 43]. Many of the studies did not justify their sample size, and non-response rates were rarely reported. Furthermore, the representativeness of some study populations was negatively affected by suboptimal sampling strategies [24, 29, 36, 46, 48, 49, 52, 53, 59]. Many of the included studies did not employ comparison groups, making it difficult to interpret whether a particular factor was more likely to influence an outcome in older populations than in the general population. Of the studies that did include a comparison group, the selection process was often poorly justified [36, 52]. Inadequate justification was also given for the selection of particular outcome measures. Among the qualitative studies, a superficial engagement with the role of the researcher and their subjectivity was a common weakness. The scores for the quality appraisal of individual studies are given in Tables 2 and the detailed results provided in Additional file 3.

## Discussion

This is the first systematic review to examine the evidence related to the specific health needs and vulnerabilities of older populations affected by humanitarian crisis in LMICs. The majority of the 36 studies meeting eligibility criteria were cross-sectional in design, restricting our ability to imply causation between vulnerability factors and health outcomes. In light of the limited breadth and quality of evidence, the following findings should be treated with caution.

This review identified that older age, female gender, socio-economic deprivation and rural residency were frequently associated with adverse health outcomes, reflecting findings from elsewhere for mental health, [60, 61] and nutrition [62]. The influence of female gender with worse health outcomes is consistent with existing research in stable settings and highlights the importance of gender-disaggregated data and further research on older women’s health needs in humanitarian crises [61, 63, 64]. The discrepancy in health outcomes between urban and rural areas is particularly concerning given that the majority of older populations in low-income countries live in rural areas [65]. Many of these risk-factors, particularly for mental health outcomes, are similar to those in all-age adult populations affected by humanitarian crises [61, 66, 67]. The limited number of studies on non-communicable diseases is also surprising given their higher burden among older people and increasing concern about non-communicable diseases in humanitarian crises [68].

The limited quantity and quality of research can be partly attributable to the inherent complexity of providing services and conducting research during humanitarian crises, but such research has been successfully undertaken with other population in humanitarian crises [69]. We identified no intervention studies on the effectiveness of existing health interventions specifically with older populations. As the context of humanitarian crises can make randomized control trials difficult to carry out (though by no means impossible [69]), quasi-experimental methods and variants such a stepped-wedge approaches could be used to gain a fuller understanding of the effectiveness (and cost-effectiveness) of health programs in meeting the needs of older populations in humanitarian crises. Routine service data could also be more effectively utilised, but this is currently hampered by the common absence of routine age-disaggregated data for older populations [70, 71]. There also needs to be considerably more qualitative research to better understand the perspectives of older populations and health care providers.

In addition to the above research recommendations, humanitarian agencies should consider ways to strengthen their work and capacity to better understand and address the health needs of older people. This includes strengthening and adhering to best practice guidelines for older people in humanitarian crises [9]. UNHCR’s Accountability Framework for Age, Gender and Diversity Mainstreaming [72] provides some information on activities for older people but much more detailed and rigorous data reporting is required. This necessitates the collection of age disaggregated routine data (as done by the Office of U.S. Foreign Disaster Assistance (OFDA) which requests disaggregated data for older age groups of 50–59 and then 60+) as well as specific data on the health needs of older people. Other activities include more training and sensitisation for humanitarian health workers on detecting and reporting the health needs of older people. This all requires substantially greater financial investment given the negligible number of funded aid projects specifically for older people in humanitarian crises. For example, of 16,221 humanitarian projects implemented between 2010 and 2014, only 74 projects were funded which included at least one activity specifically targeting older people [7].

This review has highlighted considerable weaknesses in the quantity and quality of research on the health needs of older people in humanitarian crises. While recognising the inherent constraints of humanitarian settings, the lack of research does suggest low levels of awareness and prioritisation of the needs of older populations among the heath care actors and researchers in humanitarian crises.

### Limitations

For the quality review, the NOS does not employ weighted scores for different categories and so studies can receive a strong score while still failing to consider important factors such as the representativeness of the study sample. Bivariate results were extracted where multivariate analysis was not conducted, and so these do not adjust for potential confounders.

## Conclusions

The findings from this review suggest low levels of awareness and appreciation of the needs of older populations among humanitarian heath care actors and researchers. The breadth and depth of evidence should be urgently strengthened in order to better understand the health needs of older populations and the effectiveness and appropriateness of health interventions in meeting these needs.

## Additional files

Additional file 1: Complete search terms. (DOCX 49 kb)

Additional file 2: PRISMA checklist. (DOCX 17 kb)

Additional file 3: Detailed Quality Appraisal Results. (DOCX 23 kb)
