# Supplementary material for: Health needs of older populations affected by humanitarian crises in low- and middle-income countries: a systematic review
Source: Confl Health. 2017 Dec 11;11:29. doi: 10.1186/s13031-017-0133-x (PMC5724338; doi:10.1186/s13031-017-0133-x)
Supplement: Supplementary file 1 — Complete search terms. (DOCX 49 kb) [file 13031_2017_133_MOESM1_ESM.docx]

# Additional file 1: complete search terms

## EMBASE (N=1836)

| 1. developing country/ |
| --- |
| 2. ((developing or less* developed or under developed or underdeveloped or middle income or low* income or underserved or under served or deprived or poor*) adj (economy or economies)).ti,ab. |
| 3. ((developing or less* developed or under developed or underdeveloped or middle income or low* income or underserved or under served or deprived or poor*) adj (countr* or nation? or population? or world)).ti,ab. |
| 4. (low* adj (gdp or gnp or gross domestic or gross national)).ti,ab. |
| 5. (low adj3 middle adj3 countr*).ti,ab. |
| 6. (lmic or lmics or third world or lami countr*).ti,ab. |
| 7. transitional countr*.ti,ab. |
| 8. or/1-7 |
| 9. Cambodia/ |
| 10. (cambodia or khmer republic).mp. |
| 11. North Korea/ |
| 12. (north korea or (democratic people* republic adj2 korea)).mp. |
| 13. Myanmar/ |
| 14. (myanmar or burma).mp. |
| 15. or/9-14 |
| 16. Tajikistan/ |
| 17. (tajikistan or tadzhik or tadzhikistan or tajikistan).mp. |
| 18. or/16-17 |
| 19. Haiti/ |
| 20. Haiti.mp. |
| 21. or/19-20 |
| 22. Afghanistan/ |
| 23. Afghanistan.mp. |
| 24. Bangladesh/ |
| 25. Bangladesh.mp. |
| 26. Nepal/ |
| 27. Nepal.mp. |
| 28. or/22-27 |
| 29. Benin/ |
| 30. (Benin or Dahomey).mp. |
| 31. Burkina Faso/ |
| 32. (Burkina Faso or Burkina Fasso or Upper Volta).mp. |
| 33. Burundi/ |
| 34. Burundi.mp. |
| 35. Central African Republic/ |
| 36. (Central African Republic or Ubangi-Shari).mp. |
| 37. Chad/ |
| 38. Chad.mp. |
| 39. Comoros/ |
| 40. (Comoros or Comoro Islands or Mayotte or Iles Comores).mp. |
| 41. Democratic Republic Congo/ |
| 42. ((democratic republic adj2 congo) or belgian congo or zaire).mp. |
| 43. Eritrea/ |
| 44. Eritrea.mp. |
| 45. Ethiopia/ |
| 46. Ethiopia.mp. |
| 47. Gambia/ |
| 48. Gambia.mp. |
| 49. Guinea/ |
| 50. (Guinea not (New Guinea or Guinea Pig* or Guinea Fowl)).mp. |
| 51. Guinea-Bissau/ |
| 52. (Guinea-Bissau or Portuguese Guinea).mp. |
| 53. Kenya/ |
| 54. Kenya.mp. |
| 55. Liberia/ |
| 56. Liberia.mp. |
| 57. Madagascar/ |
| 58. (Madagascar or Malagasy Republic).mp. |
| 59. Malawi/ |
| 60. (Malawi or Nyasaland).mp. |
| 61. Mali/ |
| 62. Mali.mp. |
| 63. Mozambique/ |
| 64. (Mozambique or Portuguese East Africa).mp. |
| 65. Niger/ |
| 66. (Niger not (Aspergillus or Peptococcus or Schizothorax or Cruciferae or Gobius or Lasius or Agelastes or Melanosuchus or radish or Parastromateus or Orius or Apergillus or Parastromateus or Stomoxys)).mp. |
| 67. Rwanda/ |
| 68. (Rwanda or Ruanda).mp. |
| 69. Sierra Leone/ |
| 70. Sierra Leone.mp. |
| 71. Somalia/ |
| 72. Somalia.mp. |
| 73. Tanzania/ |
| 74. (Tanzania or Zanzibar).mp. |
| 75. Togo/ |
| 76. (Togo or Togolese Republic).mp. |
| 77. Uganda/ |
| 78. Uganda.mp. |
| 79. Zimbabwe/ |
| 80. (Zimbabwe or Rhodesia).mp. |
| 81. or/29-80 |
| 82. Indonesia/ |
| 83. indonesia.mp. |
| 84. kiribati/ |
| 85. Kiribati.mp. |
| 86. "Federated States of Micronesia"/ |
| 87. micronesia.mp. |
| 88. Laos/ |
| 89. (laos or (lao adj2 democratic republic)).mp. |
| 90. marshall islands/ |
| 91. marshall island*.mp. |
| 92. Mongolia/ |
| 93. (mongolia or mongolian people* republic).mp. |
| 94. Papua New Guinea/ |
| 95. Papua New Guinea.mp. |
| 96. Philippines/ |
| 97. Philippines.mp. |
| 98. Samoa/ |
| 99. samoa.mp. |
| 100. solomon islands/ |
| 101. Solomon Islands.mp. |
| 102. Melanesia/ |
| 103. melanesia.mp. |
| 104. Timor-Leste/ |
| 105. (Timor-Leste or East Timor).mp. |
| 106. Vanuatu/ |
| 107. Vanuatu.mp. |
| 108. Viet Nam/ |
| 109. (Viet Nam or Vietnam).mp. |
| 110. or/82-109 |
| 111. Armenia/ |
| 112. Armenia.mp. |
| 113. "Georgia (republic)"/ |
| 114. (georgia not (georgia adj3 state)).mp. |
| 115. "yugoslavia (pre-1992)"/ or yugoslavia/ |
| 116. Kosovo/ |
| 117. kosovo.mp. |
| 118. Kyrgyzstan/ |
| 119. (kyrgyzstan or kyrgyz republic or kirghizia or kirghiz).mp. |
| 120. Moldova/ |
| 121. Moldova.mp. |
| 122. Ukraine/ |
| 123. Ukraine.mp. |
| 124. Uzbekistan/ |
| 125. Uzbekistan.mp. |
| 126. or/111-125 |
| 127. Bolivia/ |
| 128. Bolivia.mp. |
| 129. El Salvador/ |
| 130. salvador.mp. |
| 131. Guatemala/ |
| 132. Guatemala.mp. |
| 133. Guyana/ |
| 134. Guyana.mp. |
| 135. Honduras/ |
| 136. Honduras.mp. |
| 137. Nicaragua/ |
| 138. Nicaragua.mp. |
| 139. Paraguay/ |
| 140. Paraguay.mp. |
| 141. or/127-140 |
| 142. Djibouti/ |
| 143. (Djibouti or French Somaliland).mp. |
| 144. Egypt/ |
| 145. Egypt.mp. |
| 146. Morocco/ |
| 147. Morocco.mp. |
| 148. Syrian Arab Republic/ |
| 149. (Syria or Syrian Arab Republic).mp. |
| 150. Gaza.mp. |
| 151. Yemen/ |
| 152. Yemen.mp. |
| 153. or/142-152 |
| 154. Bhutan/ |
| 155. Bhutan.mp. |
| 156. India/ |
| 157. India.mp. |
| 158. Pakistan/ |
| 159. Pakistan.mp. |
| 160. Sri Lanka/ |
| 161. (Sri Lanka or Ceylon).mp. |
| 162. or/154-161 |
| 163. Cameroon/ |
| 164. Cameroon.mp. |
| 165. Cape Verde/ |
| 166. (Cape Verde or Cabo Verde).mp. |
| 167. Congo/ |
| 168. (congo not ((democratic republic adj3 congo) or congo red or crimean-congo)).mp. |
| 169. Cote d'Ivoire/ |
| 170. (Cote d'Ivoire or Ivory Coast).mp. |
| 171. Ghana/ |
| 172. (Ghana or Gold Coast).mp. |
| 173. Lesotho/ |
| 174. (Lesotho or Basutoland).mp. |
| 175. Mauritania/ |
| 176. Mauritania.mp. |
| 177. Nigeria/ |
| 178. Nigeria.mp. |
| 179. "sao tome and principe"/ |
| 180. (sao tome adj2 principe).mp. |
| 181. Senegal/ |
| 182. Senegal.mp. |
| 183. Sudan/ |
| 184. Sudan.mp. |
| 185. Swaziland/ |
| 186. Swaziland.mp. |
| 187. Zambia/ |
| 188. (Zambia or Northern Rhodesia).mp. |
| 189. or/163-188 |
| 190. American Samoa/ |
| 191. american samoa.mp. |
| 192. China/ |
| 193. (china or (chinese adj3 republic)).mp. |
| 194. Fiji/ |
| 195. fiji.mp. |
| 196. Malaysia/ |
| 197. malaysia.mp. |
| 198. marshall islands/ |
| 199. marshall islands.mp. |
| 200. Palau/ |
| 201. Palau.mp. |
| 202. Thailand/ |
| 203. (Thailand or Siam).mp. |
| 204. tuvalu/ |
| 205. Tuvalu.mp. |
| 206. or/190-205 |
| 207. Albania/ |
| 208. Albania.mp. |
| 209. Azerbaijan/ |
| 210. Azerbaijan.mp. |
| 211. Belarus/ |
| 212. (belarus or byelarus or belorussia).mp. |
| 213. "Bosnia and Herzegovina"/ |
| 214. bosnia.mp. |
| 215. Bulgaria/ |
| 216. Bulgaria.mp. |
| 217. Hungary/ |
| 218. hungary.mp. |
| 219. Kazakhstan/ |
| 220. (Kazakhstan or kazakh).mp. |
| 221. "Macedonia (republic)"/ |
| 222. Macedonia.mp. |
| 223. "Montenegro (republic)"/ |
| 224. Montenegro.mp. |
| 225. Romania/ |
| 226. Romania.mp. |
| 227. Serbia/ |
| 228. serbia.mp. |
| 229. "Turkey (republic)"/ |
| 230. turkey.mp. not "turkey (bird)"/ |
| 231. Turkmenistan/ |
| 232. Turkmenistan.mp. |
| 233. or/207-232 |
| 234. Argentina/ |
| 235. Argentina.mp. |
| 236. Belize/ |
| 237. (Belize or British Honduras).mp. |
| 238. Brazil/ |
| 239. Brazil.mp. |
| 240. Colombia/ |
| 241. Colombia.mp. |
| 242. Costa Rica/ |
| 243. Costa Rica.mp. |
| 244. Cuba/ |
| 245. Cuba.mp. |
| 246. Dominica/ |
| 247. Dominica.mp. |
| 248. Dominican Republic/ |
| 249. Dominican Republic.mp. |
| 250. Ecuador/ |
| 251. Ecuador.mp. |
| 252. Grenada/ |
| 253. Grenada.mp. |
| 254. Jamaica/ |
| 255. Jamaica.mp. |
| 256. Mexico/ |
| 257. Mexico.mp. |
| 258. Panama/ |
| 259. Panama.mp. |
| 260. Peru/ |
| 261. Peru.mp. |
| 262. Saint Lucia/ |
| 263. (St Lucia or Saint Lucia).mp. |
| 264. "Saint Vincent and the Grenadines"/ |
| 265. Grenadines.mp. |
| 266. Suriname/ |
| 267. Suriname.mp. |
| 268. Venezuela/ |
| 269. Venezuela.mp. |
| 270. or/234-269 |
| 271. Algeria/ |
| 272. Algeria.mp. |
| 273. Iran/ |
| 274. Iran.mp. |
| 275. Iraq/ |
| 276. Iraq.mp. |
| 277. Jordan/ |
| 278. Jordan.mp. |
| 279. Lebanon/ |
| 280. Lebanon.mp. |
| 281. Libyan Arab Jamahiriya/ |
| 282. (Libya or libyan arab jamahiriya).mp. |
| 283. Tunisia/ |
| 284. Tunisia.mp. |
| 285. or/271-284 |
| 286. maldives/ |
| 287. Maldives.mp. |
| 288. or/286-287 |
| 289. Angola/ |
| 290. Angola.mp. |
| 291. Botswana/ |
| 292. (Botswana or Bechuanaland or Kalahari).mp. |
| 293. Gabon/ |
| 294. (Gabon or Gabonese Republic).mp. |
| 295. Mauritius/ |
| 296. (Mauritius or Agalega Islands).mp. |
| 297. Namibia/ |
| 298. Namibia.mp. |
| 299. Seychelles/ |
| 300. Seychelles.mp. |
| 301. South Africa/ |
| 302. South Africa.mp. |
| 303. or/289-302 |
| 304. 8 or 15 or 18 or 21 or 81 or 110 or 126 or 141 or 153 or 162 or 189 or 206 or 233 or 270 or 285 or 288 or 303 |
| 305. exp Disasters/ |
| 306. exp Relief Work/ |
| 307. exp Rescue Work/ |
| 308. exp Emergencies/ |
| 309. exp Disaster Medicine/ |
| 310. exp Mass Casualty Incidents/ |
| 311. exp Emergency Responders/ |
| 312. (humanitarian adj2 (crisis or crises or relief or response or agenc*)).tw. |
| 313. humanitarian.tw. |
| 314. (disaster adj3 (relief or plan$)).tw. |
| 315. ((relief or aid) adj2 work$).tw. |
| 316. Refugees.tw. |
| 317. (refugee or evacuee or evacuated).tw. |
| 318. exp War/ |
| 319. War.tw. |
| 320. ((armed or zone) adj2 conflict$).tw. |
| 321. (conflict affected adj3 (population$ or person$ or communit$)).tw. |
| 322. exp avalanches/ or exp earthquakes/ or exp landslides/ or exp tidal waves/ or exp tsunamis/ or exp volcanic eruptions/ |
| 323. exp cyclonic storms/ or exp droughts/ or exp floods/ or exp tidal waves/ |
| 324. typhoon*.tw. |
| 325. hurricane*.tw. |
| 326. cyclone*.tw. |
| 327. (avalanche$ or earthquake$ or flood or floods or flooding or flooded or landslide$ or tsunami$).tw. |
| 328. (disaster adj2 (natural or victim)).tw. |
| 329. drought$.tw. |
| 330. exp Starvation/ |
| 331. (starvation or famine$).tw. |
| 332. 305 or 306 or 307 or 308 or 309 or 310 or 311 or 312 or 313 or 314 or 315 or 316 or 317 or 318 or 319 or 320 or 321 or 322 or 323 or 324 or 325 or 326 or 327 or 328 or 329 or 330 or 331 |
| 333. exp geriatrics/ |
| 334. elderly.ti,ab. |
| 335. elder.ti,ab. |
| 336. aging.ti,ab. |
| 337. older adult*.ti,ab. |
| 338. older people.ti,ab. |
| 339. older patient*.ti,ab. |
| 340. older women.ti,ab. |
| 341. older men.ti,ab. |
| 342. older individual*.ti,ab. |
| 343. geriatric*.ti,ab. |
| 344. gerontology.ti,ab. |
| 345. senior citizen.ti,ab. |
| 346. senior citizens.ti,ab. |
| 347. seniors.ti,ab. |
| 348. septuagenarian*.ti,ab. |
| 349. octogenarian*.ti,ab. |
| 350. nonagenarian*.ti,ab. |
| 351. exp aged/ |
| 352. elders.ti,ab. |
| 353. older person*.ti,ab. |
| 354. 333 or 334 or 335 or 336 or 337 or 338 or 339 or 340 or 341 or 342 or 343 or 344 or 345 or 346 or 347 or 348 or 349 or 350 or 351 or 352 or 353 |
| 355. 304 and 332 and 354 |

##

## Medline (N=1911)

| 1. ((developing or less* developed or under developed or underdeveloped or middle income or low* income or underserved or under served or deprived or poor*) adj (economy or economies)).ti,ab. |
| --- |
| 2. ((developing or less* developed or under developed or underdeveloped or middle income or low* income or underserved or under served or deprived or poor*) adj (countr* or nation? or population? or world)).ti,ab. |
| 3. (low* adj (gdp or gnp or gross domestic or gross national)).ti,ab. |
| 4. (low adj3 middle adj3 countr*).ti,ab. |
| 5. (lmic or lmics or third world or lami countr*).ti,ab. |
| 6. transitional countr*.ti,ab. |
| 7. Developing Countries/ |
| 8. or/1-7 |
| 9. Cambodia/ |
| 10. cambodia.mp. [mp=title, abstract, original title, name of substance word, subject heading word, keyword heading word, protocol supplementary concept word, rare disease supplementary concept word, unique identifier] |
| 11. "Democratic People's Republic of Korea"/ |
| 12. (north korea or (democratic people* republic adj2 korea)).mp. [mp=title, abstract, original title, name of substance word, subject heading word, keyword heading word, protocol supplementary concept word, rare disease supplementary concept word, unique identifier] |
| 13. Myanmar/ |
| 14. (myanmar or burma).mp. [mp=title, abstract, original title, name of substance word, subject heading word, keyword heading word, protocol supplementary concept word, rare disease supplementary concept word, unique identifier] |
| 15. or/9-14 |
| 16. Indonesia/ |
| 17. indonesia.mp. [mp=title, abstract, original title, name of substance word, subject heading word, keyword heading word, protocol supplementary concept word, rare disease supplementary concept word, unique identifier] |
| 18. Micronesia/ |
| 19. Kiribati.mp. [mp=title, abstract, original title, name of substance word, subject heading word, keyword heading word, protocol supplementary concept word, rare disease supplementary concept word, unique identifier] |
| 20. Laos/ |
| 21. (laos or (lao adj1 democratic republic)).mp. [mp=title, abstract, original title, name of substance word, subject heading word, keyword heading word, protocol supplementary concept word, rare disease supplementary concept word, unique identifier] |
| 22. marshall island*.mp. [mp=title, abstract, original title, name of substance word, subject heading word, keyword heading word, protocol supplementary concept word, rare disease supplementary concept word, unique identifier] |
| 23. Mongolia/ |
| 24. mongolia.mp. [mp=title, abstract, original title, name of substance word, subject heading word, keyword heading word, protocol supplementary concept word, rare disease supplementary concept word, unique identifier] |
| 25. Papua New Guinea/ |
| 26. Papua New Guinea.mp. [mp=title, abstract, original title, name of substance word, subject heading word, keyword heading word, protocol supplementary concept word, rare disease supplementary concept word, unique identifier] |
| 27. Philippines/ |
| 28. Philippines.mp. [mp=title, abstract, original title, name of substance word, subject heading word, keyword heading word, protocol supplementary concept word, rare disease supplementary concept word, unique identifier] |
| 29. samoa/ or "independent state of samoa"/ |
| 30. samoa.mp. [mp=title, abstract, original title, name of substance word, subject heading word, keyword heading word, protocol supplementary concept word, rare disease supplementary concept word, unique identifier] |
| 31. Melanesia/ |
| 32. Solomon Islands.mp. [mp=title, abstract, original title, name of substance word, subject heading word, keyword heading word, protocol supplementary concept word, rare disease supplementary concept word, unique identifier] |
| 33. Timor-Leste.mp. |
| 34. Vanuatu/ |
| 35. Vanuatu.mp. [mp=title, abstract, original title, name of substance word, subject heading word, keyword heading word, protocol supplementary concept word, rare disease supplementary concept word, unique identifier] |
| 36. Vietnam/ |
| 37. Vietnam.mp. [mp=title, abstract, original title, name of substance word, subject heading word, keyword heading word, protocol supplementary concept word, rare disease supplementary concept word, unique identifier] |
| 38. or/16-37 |
| 39. American Samoa/ |
| 40. american samoa.mp. [mp=title, abstract, original title, name of substance word, subject heading word, keyword heading word, protocol supplementary concept word, rare disease supplementary concept word, unique identifier] |
| 41. exp China/ |
| 42. china.mp. [mp=title, abstract, original title, name of substance word, subject heading word, keyword heading word, protocol supplementary concept word, rare disease supplementary concept word, unique identifier] |
| 43. Fiji/ |
| 44. fiji.mp. [mp=title, abstract, original title, name of substance word, subject heading word, keyword heading word, protocol supplementary concept word, rare disease supplementary concept word, unique identifier] |
| 45. Malaysia/ |
| 46. malaysia.mp. [mp=title, abstract, original title, name of substance word, subject heading word, keyword heading word, protocol supplementary concept word, rare disease supplementary concept word, unique identifier] |
| 47. marshall islands.mp. [mp=title, abstract, original title, name of substance word, subject heading word, keyword heading word, protocol supplementary concept word, rare disease supplementary concept word, unique identifier] |
| 48. Palau/ |
| 49. Palau.mp. [mp=title, abstract, original title, name of substance word, subject heading word, keyword heading word, protocol supplementary concept word, rare disease supplementary concept word, unique identifier] |
| 50. Thailand/ |
| 51. Thailand.mp. [mp=title, abstract, original title, name of substance word, subject heading word, keyword heading word, protocol supplementary concept word, rare disease supplementary concept word, unique identifier] |
| 52. Tuvalu.mp. [mp=title, abstract, original title, name of substance word, subject heading word, keyword heading word, protocol supplementary concept word, rare disease supplementary concept word, unique identifier] |
| 53. or/39-52 |
| 54. Tajikistan/ |
| 55. (tajikistan or tadzhik or tadzhikistan or tajikistan).mp. [mp=title, abstract, original title, name of substance word, subject heading word, keyword heading word, protocol supplementary concept word, rare disease supplementary concept word, unique identifier] |
| 56. or/54-55 |
| 57. Armenia/ |
| 58. Armenia.mp. [mp=title, abstract, original title, name of substance word, subject heading word, keyword heading word, protocol supplementary concept word, rare disease supplementary concept word, unique identifier] |
| 59. "Georgia (Republic)"/ |
| 60. Yugoslavia/ |
| 61. Kosovo.mp. [mp=title, abstract, original title, name of substance word, subject heading word, keyword heading word, protocol supplementary concept word, rare disease supplementary concept word, unique identifier] |
| 62. Kyrgyzstan/ |
| 63. (kyrgyzstan or kyrgyz republic or kirghizia or kirghiz).mp. [mp=title, abstract, original title, name of substance word, subject heading word, keyword heading word, protocol supplementary concept word, rare disease supplementary concept word, unique identifier] |
| 64. Moldova/ |
| 65. Moldova.mp. [mp=title, abstract, original title, name of substance word, subject heading word, keyword heading word, protocol supplementary concept word, rare disease supplementary concept word, unique identifier] |
| 66. Ukraine/ |
| 67. Ukraine.mp. [mp=title, abstract, original title, name of substance word, subject heading word, keyword heading word, protocol supplementary concept word, rare disease supplementary concept word, unique identifier] |
| 68. Uzbekistan/ |
| 69. Uzbekistan.mp. [mp=title, abstract, original title, name of substance word, subject heading word, keyword heading word, protocol supplementary concept word, rare disease supplementary concept word, unique identifier] |
| 70. or/57-69 |
| 71. Albania/ |
| 72. Albania.mp. [mp=title, abstract, original title, name of substance word, subject heading word, keyword heading word, protocol supplementary concept word, rare disease supplementary concept word, unique identifier] |
| 73. Azerbaijan/ |
| 74. Azerbaijan.mp. [mp=title, abstract, original title, name of substance word, subject heading word, keyword heading word, protocol supplementary concept word, rare disease supplementary concept word, unique identifier] |
| 75. "Republic of Belarus"/ |
| 76. (belarus or byelarus or belorussia).mp. [mp=title, abstract, original title, name of substance word, subject heading word, keyword heading word, protocol supplementary concept word, rare disease supplementary concept word, unique identifier] |
| 77. Bosnia-Herzegovina/ |
| 78. bosnia.mp. [mp=title, abstract, original title, name of substance word, subject heading word, keyword heading word, protocol supplementary concept word, rare disease supplementary concept word, unique identifier] |
| 79. Bulgaria/ |
| 80. Bulgaria.mp. [mp=title, abstract, original title, name of substance word, subject heading word, keyword heading word, protocol supplementary concept word, rare disease supplementary concept word, unique identifier] |
| 81. Hungary/ |
| 82. hungary.mp. [mp=title, abstract, original title, name of substance word, subject heading word, keyword heading word, protocol supplementary concept word, rare disease supplementary concept word, unique identifier] |
| 83. Kazakhstan/ |
| 84. (Kazakhstan or kazakh).mp. [mp=title, abstract, original title, name of substance word, subject heading word, keyword heading word, protocol supplementary concept word, rare disease supplementary concept word, unique identifier] |
| 85. "Macedonia (Republic)"/ |
| 86. Macedonia.mp. [mp=title, abstract, original title, name of substance word, subject heading word, keyword heading word, protocol supplementary concept word, rare disease supplementary concept word, unique identifier] |
| 87. Montenegro/ |
| 88. Montenegro.mp. [mp=title, abstract, original title, name of substance word, subject heading word, keyword heading word, protocol supplementary concept word, rare disease supplementary concept word, unique identifier] |
| 89. Romania/ |
| 90. Romania.mp. [mp=title, abstract, original title, name of substance word, subject heading word, keyword heading word, protocol supplementary concept word, rare disease supplementary concept word, unique identifier] |
| 91. Serbia/ |
| 92. serbia.mp. [mp=title, abstract, original title, name of substance word, subject heading word, keyword heading word, protocol supplementary concept word, rare disease supplementary concept word, unique identifier] |
| 93. Turkey/ |
| 94. turkey.mp. not animal/ [mp=title, abstract, original title, name of substance word, subject heading word, keyword heading word, protocol supplementary concept word, rare disease supplementary concept word, unique identifier] |
| 95. Turkmenistan/ |
| 96. Turkmenistan.mp. [mp=title, abstract, original title, name of substance word, subject heading word, keyword heading word, protocol supplementary concept word, rare disease supplementary concept word, unique identifier] |
| 97. or/71-96 |
| 98. Haiti/ |
| 99. Haiti.mp. [mp=title, abstract, original title, name of substance word, subject heading word, keyword heading word, protocol supplementary concept word, rare disease supplementary concept word, unique identifier] |
| 100. 98 or 99 |
| 101. Bolivia/ |
| 102. Bolivia.mp. [mp=title, abstract, original title, name of substance word, subject heading word, keyword heading word, protocol supplementary concept word, rare disease supplementary concept word, unique identifier] |
| 103. El Salvador/ |
| 104. El Salvador.mp. [mp=title, abstract, original title, name of substance word, subject heading word, keyword heading word, protocol supplementary concept word, rare disease supplementary concept word, unique identifier] |
| 105. Guatemala/ |
| 106. Guatemala.mp. [mp=title, abstract, original title, name of substance word, subject heading word, keyword heading word, protocol supplementary concept word, rare disease supplementary concept word, unique identifier] |
| 107. Guyana/ |
| 108. Guyana.mp. [mp=title, abstract, original title, name of substance word, subject heading word, keyword heading word, protocol supplementary concept word, rare disease supplementary concept word, unique identifier] |
| 109. Honduras/ |
| 110. Honduras.mp. [mp=title, abstract, original title, name of substance word, subject heading word, keyword heading word, protocol supplementary concept word, rare disease supplementary concept word, unique identifier] |
| 111. Nicaragua/ |
| 112. Nicaragua.mp. [mp=title, abstract, original title, name of substance word, subject heading word, keyword heading word, protocol supplementary concept word, rare disease supplementary concept word, unique identifier] |
| 113. Paraguay/ |
| 114. Paraguay.mp. [mp=title, abstract, original title, name of substance word, subject heading word, keyword heading word, protocol supplementary concept word, rare disease supplementary concept word, unique identifier] |
| 115. or/101-114 |
| 116. Argentina/ |
| 117. Argentina.mp. [mp=title, abstract, original title, name of substance word, subject heading word, keyword heading word, protocol supplementary concept word, rare disease supplementary concept word, unique identifier] |
| 118. Belize/ |
| 119. Belize.mp. [mp=title, abstract, original title, name of substance word, subject heading word, keyword heading word, protocol supplementary concept word, rare disease supplementary concept word, unique identifier] |
| 120. Brazil/ |
| 121. Brazil.mp. [mp=title, abstract, original title, name of substance word, subject heading word, keyword heading word, protocol supplementary concept word, rare disease supplementary concept word, unique identifier] |
| 122. Colombia/ |
| 123. Colombia.mp. [mp=title, abstract, original title, name of substance word, subject heading word, keyword heading word, protocol supplementary concept word, rare disease supplementary concept word, unique identifier] |
| 124. Costa Rica/ |
| 125. Costa Rica.mp. [mp=title, abstract, original title, name of substance word, subject heading word, keyword heading word, protocol supplementary concept word, rare disease supplementary concept word, unique identifier] |
| 126. Cuba/ |
| 127. Cuba.mp. [mp=title, abstract, original title, name of substance word, subject heading word, keyword heading word, protocol supplementary concept word, rare disease supplementary concept word, unique identifier] |
| 128. Dominica/ |
| 129. Dominica.mp. [mp=title, abstract, original title, name of substance word, subject heading word, keyword heading word, protocol supplementary concept word, rare disease supplementary concept word, unique identifier] |
| 130. Dominican Republic/ |
| 131. Dominican Republic.mp. [mp=title, abstract, original title, name of substance word, subject heading word, keyword heading word, protocol supplementary concept word, rare disease supplementary concept word, unique identifier] |
| 132. Ecuador/ |
| 133. Ecuador.mp. [mp=title, abstract, original title, name of substance word, subject heading word, keyword heading word, protocol supplementary concept word, rare disease supplementary concept word, unique identifier] |
| 134. Grenada/ |
| 135. Grenada.mp. [mp=title, abstract, original title, name of substance word, subject heading word, keyword heading word, protocol supplementary concept word, rare disease supplementary concept word, unique identifier] |
| 136. Jamaica/ |
| 137. Jamaica.mp. [mp=title, abstract, original title, name of substance word, subject heading word, keyword heading word, protocol supplementary concept word, rare disease supplementary concept word, unique identifier] |
| 138. Mexico/ |
| 139. Mexico.mp. [mp=title, abstract, original title, name of substance word, subject heading word, keyword heading word, protocol supplementary concept word, rare disease supplementary concept word, unique identifier] |
| 140. exp Panama/ |
| 141. Panama.mp. [mp=title, abstract, original title, name of substance word, subject heading word, keyword heading word, protocol supplementary concept word, rare disease supplementary concept word, unique identifier] |
| 142. Peru/ |
| 143. Peru.mp. [mp=title, abstract, original title, name of substance word, subject heading word, keyword heading word, protocol supplementary concept word, rare disease supplementary concept word, unique identifier] |
| 144. Saint Lucia/ |
| 145. (St Lucia or Saint Lucia).mp. [mp=title, abstract, original title, name of substance word, subject heading word, keyword heading word, protocol supplementary concept word, rare disease supplementary concept word, unique identifier] |
| 146. "Saint Vincent and the Grenadines"/ |
| 147. Grenadines.mp. [mp=title, abstract, original title, name of substance word, subject heading word, keyword heading word, protocol supplementary concept word, rare disease supplementary concept word, unique identifier] |
| 148. Suriname/ |
| 149. Suriname.mp. [mp=title, abstract, original title, name of substance word, subject heading word, keyword heading word, protocol supplementary concept word, rare disease supplementary concept word, unique identifier] |
| 150. Venezuela/ |
| 151. Venezuela.mp. [mp=title, abstract, original title, name of substance word, subject heading word, keyword heading word, protocol supplementary concept word, rare disease supplementary concept word, unique identifier] |
| 152. Djibouti/ |
| 153. (Djibouti or French Somaliland).mp. [mp=title, abstract, original title, name of substance word, subject heading word, keyword heading word, protocol supplementary concept word, rare disease supplementary concept word, unique identifier] |
| 154. Egypt/ |
| 155. Egypt.mp. [mp=title, abstract, original title, name of substance word, subject heading word, keyword heading word, protocol supplementary concept word, rare disease supplementary concept word, unique identifier] |
| 156. Morocco/ |
| 157. Morocco.mp. [mp=title, abstract, original title, name of substance word, subject heading word, keyword heading word, protocol supplementary concept word, rare disease supplementary concept word, unique identifier] |
| 158. Syria/ |
| 159. (Syria or Syrian Arab Republic).mp. [mp=title, abstract, original title, name of substance word, subject heading word, keyword heading word, protocol supplementary concept word, rare disease supplementary concept word, unique identifier] |
| 160. Gaza.mp. [mp=title, abstract, original title, name of substance word, subject heading word, keyword heading word, protocol supplementary concept word, rare disease supplementary concept word, unique identifier] |
| 161. Yemen/ |
| 162. Yemen.mp. [mp=title, abstract, original title, name of substance word, subject heading word, keyword heading word, protocol supplementary concept word, rare disease supplementary concept word, unique identifier] |
| 163. or/152-162 |
| 164. Algeria/ |
| 165. Algeria.mp. [mp=title, abstract, original title, name of substance word, subject heading word, keyword heading word, protocol supplementary concept word, rare disease supplementary concept word, unique identifier] |
| 166. Iran/ |
| 167. Iran.mp. [mp=title, abstract, original title, name of substance word, subject heading word, keyword heading word, protocol supplementary concept word, rare disease supplementary concept word, unique identifier] |
| 168. Iraq/ |
| 169. Iraq.mp. [mp=title, abstract, original title, name of substance word, subject heading word, keyword heading word, protocol supplementary concept word, rare disease supplementary concept word, unique identifier] |
| 170. Jordan/ |
| 171. Jordan.mp. [mp=title, abstract, original title, name of substance word, subject heading word, keyword heading word, protocol supplementary concept word, rare disease supplementary concept word, unique identifier] |
| 172. Lebanon/ |
| 173. Lebanon.mp. [mp=title, abstract, original title, name of substance word, subject heading word, keyword heading word, protocol supplementary concept word, rare disease supplementary concept word, unique identifier] |
| 174. Libya/ |
| 175. Libya.mp. [mp=title, abstract, original title, name of substance word, subject heading word, keyword heading word, protocol supplementary concept word, rare disease supplementary concept word, unique identifier] |
| 176. Tunisia/ |
| 177. Tunisia.mp. [mp=title, abstract, original title, name of substance word, subject heading word, keyword heading word, protocol supplementary concept word, rare disease supplementary concept word, unique identifier] |
| 178. or/164-177 |
| 179. Afghanistan/ |
| 180. Afghanistan.mp. [mp=title, abstract, original title, name of substance word, subject heading word, keyword heading word, protocol supplementary concept word, rare disease supplementary concept word, unique identifier] |
| 181. Bangladesh/ |
| 182. Bangladesh.mp. [mp=title, abstract, original title, name of substance word, subject heading word, keyword heading word, protocol supplementary concept word, rare disease supplementary concept word, unique identifier] |
| 183. Nepal/ |
| 184. Nepal.mp. [mp=title, abstract, original title, name of substance word, subject heading word, keyword heading word, protocol supplementary concept word, rare disease supplementary concept word, unique identifier] |
| 185. or/179-184 |
| 186. Bhutan/ |
| 187. Bhutan.mp. [mp=title, abstract, original title, name of substance word, subject heading word, keyword heading word, protocol supplementary concept word, rare disease supplementary concept word, unique identifier] |
| 188. exp India/ |
| 189. India.mp. [mp=title, abstract, original title, name of substance word, subject heading word, keyword heading word, protocol supplementary concept word, rare disease supplementary concept word, unique identifier] |
| 190. Pakistan/ |
| 191. Pakistan.mp. [mp=title, abstract, original title, name of substance word, subject heading word, keyword heading word, protocol supplementary concept word, rare disease supplementary concept word, unique identifier] |
| 192. Sri Lanka/ |
| 193. Sri Lanka.mp. [mp=title, abstract, original title, name of substance word, subject heading word, keyword heading word, protocol supplementary concept word, rare disease supplementary concept word, unique identifier] |
| 194. or/186-193 |
| 195. Indian Ocean Islands/ |
| 196. Maldives.mp. [mp=title, abstract, original title, name of substance word, subject heading word, keyword heading word, protocol supplementary concept word, rare disease supplementary concept word, unique identifier] |
| 197. or/195-196 |
| 198. Benin/ |
| 199. (Benin or Dahomey).mp. [mp=title, abstract, original title, name of substance word, subject heading word, keyword heading word, protocol supplementary concept word, rare disease supplementary concept word, unique identifier] |
| 200. Burkina Faso/ |
| 201. (Burkina Faso or Burkina Fasso or Upper Volta).mp. [mp=title, abstract, original title, name of substance word, subject heading word, keyword heading word, protocol supplementary concept word, rare disease supplementary concept word, unique identifier] |
| 202. Burundi/ |
| 203. Burundi.mp. [mp=title, abstract, original title, name of substance word, subject heading word, keyword heading word, protocol supplementary concept word, rare disease supplementary concept word, unique identifier] |
| 204. Central African Republic/ |
| 205. (Central African Republic or Ubangi-Shari).mp. [mp=title, abstract, original title, name of substance word, subject heading word, keyword heading word, protocol supplementary concept word, rare disease supplementary concept word, unique identifier] |
| 206. Chad/ |
| 207. Chad.mp. [mp=title, abstract, original title, name of substance word, subject heading word, keyword heading word, protocol supplementary concept word, rare disease supplementary concept word, unique identifier] |
| 208. Comoros/ |
| 209. (Comoros or Comoro Islands or Mayotte or Iles Comores).mp. [mp=title, abstract, original title, name of substance word, subject heading word, keyword heading word, protocol supplementary concept word, rare disease supplementary concept word, unique identifier] |
| 210. "Democratic Republic of the Congo"/ |
| 211. ((democratic republic adj2 congo) or belgian congo or zaire).mp. [mp=title, abstract, original title, name of substance word, subject heading word, keyword heading word, protocol supplementary concept word, rare disease supplementary concept word, unique identifier] |
| 212. Eritrea/ |
| 213. Eritrea.mp. [mp=title, abstract, original title, name of substance word, subject heading word, keyword heading word, protocol supplementary concept word, rare disease supplementary concept word, unique identifier] |
| 214. Ethiopia/ |
| 215. Ethiopia.mp. [mp=title, abstract, original title, name of substance word, subject heading word, keyword heading word, protocol supplementary concept word, rare disease supplementary concept word, unique identifier] |
| 216. Gambia/ |
| 217. Gambia.mp. [mp=title, abstract, original title, name of substance word, subject heading word, keyword heading word, protocol supplementary concept word, rare disease supplementary concept word, unique identifier] |
| 218. Guinea/ |
| 219. (Guinea not (New Guinea or Guinea Pig* or Guinea Fowl)).mp. [mp=title, abstract, original title, name of substance word, subject heading word, keyword heading word, protocol supplementary concept word, rare disease supplementary concept word, unique identifier] |
| 220. Guinea-Bissau/ |
| 221. (Guinea-Bissau or Portuguese Guinea).mp. [mp=title, abstract, original title, name of substance word, subject heading word, keyword heading word, protocol supplementary concept word, rare disease supplementary concept word, unique identifier] |
| 222. Kenya/ |
| 223. Kenya.mp. [mp=title, abstract, original title, name of substance word, subject heading word, keyword heading word, protocol supplementary concept word, rare disease supplementary concept word, unique identifier] |
| 224. Liberia/ |
| 225. Liberia.mp. [mp=title, abstract, original title, name of substance word, subject heading word, keyword heading word, protocol supplementary concept word, rare disease supplementary concept word, unique identifier] |
| 226. Madagascar/ |
| 227. (Madagascar or Malagasy Republic).mp. [mp=title, abstract, original title, name of substance word, subject heading word, keyword heading word, protocol supplementary concept word, rare disease supplementary concept word, unique identifier] |
| 228. Malawi/ |
| 229. (Malawi or Nyasaland).mp. [mp=title, abstract, original title, name of substance word, subject heading word, keyword heading word, protocol supplementary concept word, rare disease supplementary concept word, unique identifier] |
| 230. Mali/ |
| 231. Mali.mp. [mp=title, abstract, original title, name of substance word, subject heading word, keyword heading word, protocol supplementary concept word, rare disease supplementary concept word, unique identifier] |
| 232. Mozambique/ |
| 233. (Mozambique or Portuguese East Africa).mp. [mp=title, abstract, original title, name of substance word, subject heading word, keyword heading word, protocol supplementary concept word, rare disease supplementary concept word, unique identifier] |
| 234. Niger/ |
| 235. (Niger not (Aspergillus or Peptococcus or Schizothorax or Cruciferae or Gobius or Lasius or Agelastes or Melanosuchus or radish or Parastromateus or Orius or Apergillus or Parastromateus or Stomoxys)).mp. [mp=title, abstract, original title, name of substance word, subject heading word, keyword heading word, protocol supplementary concept word, rare disease supplementary concept word, unique identifier] |
| 236. Rwanda/ |
| 237. (Rwanda or Ruanda).mp. [mp=title, abstract, original title, name of substance word, subject heading word, keyword heading word, protocol supplementary concept word, rare disease supplementary concept word, unique identifier] |
| 238. Sierra Leone/ |
| 239. Sierra Leone.mp. |
| 240. Somalia/ |
| 241. Somalia.mp. [mp=title, abstract, original title, name of substance word, subject heading word, keyword heading word, protocol supplementary concept word, rare disease supplementary concept word, unique identifier] |
| 242. Tanzania/ |
| 243. (Tanzania or Zanzibar).mp. [mp=title, abstract, original title, name of substance word, subject heading word, keyword heading word, protocol supplementary concept word, rare disease supplementary concept word, unique identifier] |
| 244. Togo/ |
| 245. (Togo or Togolese Republic).mp. [mp=title, abstract, original title, name of substance word, subject heading word, keyword heading word, protocol supplementary concept word, rare disease supplementary concept word, unique identifier] |
| 246. Uganda/ |
| 247. Uganda.mp. [mp=title, abstract, original title, name of substance word, subject heading word, keyword heading word, protocol supplementary concept word, rare disease supplementary concept word, unique identifier] |
| 248. Zimbabwe/ |
| 249. (Zimbabwe or Rhodesia).mp. [mp=title, abstract, original title, name of substance word, subject heading word, keyword heading word, protocol supplementary concept word, rare disease supplementary concept word, unique identifier] |
| 250. or/198-249 |
| 251. Cameroon/ |
| 252. Cameroon.mp. [mp=title, abstract, original title, name of substance word, subject heading word, keyword heading word, protocol supplementary concept word, rare disease supplementary concept word, unique identifier] |
| 253. Cape Verde/ |
| 254. (Cape Verde or Cabo Verde).mp. [mp=title, abstract, original title, name of substance word, subject heading word, keyword heading word, protocol supplementary concept word, rare disease supplementary concept word, unique identifier] |
| 255. Congo/ |
| 256. (congo not ((democratic republic adj3 congo) or congo red or crimean-congo)).mp. [mp=title, abstract, original title, name of substance word, subject heading word, keyword heading word, protocol supplementary concept word, rare disease supplementary concept word, unique identifier] |
| 257. Cote d'Ivoire/ |
| 258. (Cote d'Ivoire or Ivory Coast).mp. [mp=title, abstract, original title, name of substance word, subject heading word, keyword heading word, protocol supplementary concept word, rare disease supplementary concept word, unique identifier] |
| 259. Ghana/ |
| 260. (Ghana or Gold Coast).mp. [mp=title, abstract, original title, name of substance word, subject heading word, keyword heading word, protocol supplementary concept word, rare disease supplementary concept word, unique identifier] |
| 261. Lesotho/ |
| 262. (Lesotho or Basutoland).mp. [mp=title, abstract, original title, name of substance word, subject heading word, keyword heading word, protocol supplementary concept word, rare disease supplementary concept word, unique identifier] |
| 263. Mauritania/ |
| 264. Mauritania.mp. [mp=title, abstract, original title, name of substance word, subject heading word, keyword heading word, protocol supplementary concept word, rare disease supplementary concept word, unique identifier] |
| 265. Nigeria/ |
| 266. Nigeria.mp. [mp=title, abstract, original title, name of substance word, subject heading word, keyword heading word, protocol supplementary concept word, rare disease supplementary concept word, unique identifier] |
| 267. Atlantic Islands/ |
| 268. (sao tome adj2 principe).mp. [mp=title, abstract, original title, name of substance word, subject heading word, keyword heading word, protocol supplementary concept word, rare disease supplementary concept word, unique identifier] |
| 269. Senegal/ |
| 270. Senegal.mp. [mp=title, abstract, original title, name of substance word, subject heading word, keyword heading word, protocol supplementary concept word, rare disease supplementary concept word, unique identifier] |
| 271. Sudan/ |
| 272. Sudan.mp. [mp=title, abstract, original title, name of substance word, subject heading word, keyword heading word, protocol supplementary concept word, rare disease supplementary concept word, unique identifier] |
| 273. South Sudan.mp. [mp=title, abstract, original title, name of substance word, subject heading word, keyword heading word, protocol supplementary concept word, rare disease supplementary concept word, unique identifier] |
| 274. Swaziland/ |
| 275. Swaziland.mp. [mp=title, abstract, original title, name of substance word, subject heading word, keyword heading word, protocol supplementary concept word, rare disease supplementary concept word, unique identifier] |
| 276. Zambia/ |
| 277. (Zambia or Northern Rhodesia).mp. [mp=title, abstract, original title, name of substance word, subject heading word, keyword heading word, protocol supplementary concept word, rare disease supplementary concept word, unique identifier] |
| 278. or/251-275 |
| 279. Angola/ |
| 280. Angola.mp. [mp=title, abstract, original title, name of substance word, subject heading word, keyword heading word, protocol supplementary concept word, rare disease supplementary concept word, unique identifier] |
| 281. Botswana/ |
| 282. (Botswana or Bechuanaland or Kalahari).mp. [mp=title, abstract, original title, name of substance word, subject heading word, keyword heading word, protocol supplementary concept word, rare disease supplementary concept word, unique identifier] |
| 283. Gabon/ |
| 284. (Gabon or Gabonese Republic).mp. [mp=title, abstract, original title, name of substance word, subject heading word, keyword heading word, protocol supplementary concept word, rare disease supplementary concept word, unique identifier] |
| 285. Mauritius/ |
| 286. (Mauritius or Agalega Islands).mp. [mp=title, abstract, original title, name of substance word, subject heading word, keyword heading word, protocol supplementary concept word, rare disease supplementary concept word, unique identifier] |
| 287. Namibia/ |
| 288. Namibia.mp. [mp=title, abstract, original title, name of substance word, subject heading word, keyword heading word, protocol supplementary concept word, rare disease supplementary concept word, unique identifier] |
| 289. Seychelles/ |
| 290. Seychelles.mp. [mp=title, abstract, original title, name of substance word, subject heading word, keyword heading word, protocol supplementary concept word, rare disease supplementary concept word, unique identifier] |
| 291. South Africa/ |
| 292. South Africa.mp. [mp=title, abstract, original title, name of substance word, subject heading word, keyword heading word, protocol supplementary concept word, rare disease supplementary concept word, unique identifier] |
| 293. or/279-292 |
| 294. 8 or 15 or 38 or 53 or 56 or 70 or 97 or 100 or 115 or 163 or 178 or 185 or 194 or 197 or 250 or 278 or 293 |
| 295. exp Disasters/ |
| 296. exp Relief Work/ |
| 297. exp Rescue Work/ |
| 298. exp Emergencies/ |
| 299. exp Disaster Medicine/ |
| 300. exp Mass Casualty Incidents/ |
| 301. exp Emergency Responders/ |
| 302. (humanitarian adj2 (crisis or crises or relief or response or agenc*)).tw. |
| 303. humanitarian.tw. |
| 304. (disaster adj3 (relief or plan$)).tw. |
| 305. ((relief or aid) adj2 work$).tw. |
| 306. Refugees.tw. |
| 307. (refugee or evacuee or evacuated).tw. |
| 308. exp War/ |
| 309. War.tw. |
| 310. ((armed or zone) adj2 conflict$).tw. |
| 311. (conflict affected adj3 (population$ or person$ or communit$)).tw. |
| 312. exp avalanches/ or exp earthquakes/ or exp landslides/ or exp tidal waves/ or exp tsunamis/ or exp volcanic eruptions/ |
| 313. exp cyclonic storms/ or exp droughts/ or exp floods/ or exp tidal waves/ |
| 314. typhoon*.tw. |
| 315. hurricane*.tw. |
| 316. cyclone*.tw. |
| 317. (avalanche$ or earthquake$ or flood or floods or flooding or flooded or landslide$ or tsunami$).tw. |
| 318. (disaster adj2 (natural or victim)).tw. |
| 319. drought$.tw. |
| 320. exp Starvation/ |
| 321. (starvation or famine$).tw. |
| 322. 295 or 296 or 297 or 298 or 299 or 300 or 301 or 302 or 303 or 304 or 305 or 306 or 307 or 308 or 309 or 310 or 311 or 312 or 313 or 314 or 315 or 316 or 317 or 318 or 319 or 320 or 321 |
| 323. exp geriatrics/ |
| 324. elderly.ti,ab. |
| 325. elder.ti,ab. |
| 326. aging.ti,ab. |
| 327. older adult*.ti,ab. |
| 328. older people.ti,ab. |
| 329. older patient*.ti,ab. |
| 330. older women.ti,ab. |
| 331. older men.ti,ab. |
| 332. older individual*.ti,ab. |
| 333. geriatric*.ti,ab. |
| 334. gerontology.ti,ab. |
| 335. senior citizen.ti,ab. |
| 336. senior citizens.ti,ab. |
| 337. seniors.ti,ab. |
| 338. septuagenarian*.ti,ab. |
| 339. octogenarian*.ti,ab. |
| 340. nonagenarian*.ti,ab. |
| 341. exp aged/ |
| 342. elders.ti,ab. |
| 343. older person*.ti,ab. |
| 344. 323 or 324 or 325 or 326 or 327 or 328 or 329 or 330 or 331 or 332 or 333 or 334 or 335 or 336 or 337 or 338 or 339 or 340 or 341 or 342 or 343 |
| 345. 294 and 322 and 344 |

## Global Health (N=267)

| 1. exp Disasters/ |
| --- |
| 2. exp Relief Work/ |
| 3. exp Rescue Work/ |
| 4. exp Emergencies/ |
| 5. exp Disaster Medicine/ |
| 6. exp Mass Casualty Incidents/ |
| 7. exp Emergency Responders/ |
| 8. (humanitarian adj2 (crisis or crises or relief or response or agenc*)).tw. |
| 9. humanitarian.tw. |
| 10. (disaster adj3 (relief or plan$)).tw. |
| 11. ((relief or aid) adj2 work$).tw. |
| 12. Refugees.tw. |
| 13. (refugee or evacuee or evacuated).tw. |
| 14. exp War/ |
| 15. War.tw. |
| 16. ((armed or zone) adj2 conflict$).tw. |
| 17. (conflict affected adj3 (population$ or person$ or communit$)).tw. |
| 18. exp avalanches/ or exp earthquakes/ or exp landslides/ or exp tidal waves/ or exp tsunamis/ or exp volcanic eruptions/ |
| 19. exp cyclonic storms/ or exp droughts/ or exp floods/ or exp tidal waves/ |
| 20. typhoon*.tw. |
| 21. hurricane*.tw. |
| 22. cyclone*.tw. |
| 23. (avalanche$ or earthquake$ or flood or floods or flooding or flooded or landslide$ or tsunami$).tw. |
| 24. (disaster adj2 (natural or victim)).tw. |
| 25. drought$.tw. |
| 26. exp Starvation/ |
| 27. (starvation or famine$).tw. |
| 28. 1 or 2 or 3 or 4 or 5 or 6 or 7 or 8 or 9 or 10 or 11 or 12 or 13 or 14 or 15 or 16 or 17 or 18 or 19 or 20 or 21 or 22 or 23 or 24 or 25 or 26 or 27 |
| 29. exp geriatrics/ |
| 30. elderly.ti,ab. |
| 31. elder.ti,ab. |
| 32. aging.ti,ab. |
| 33. older adult*.ti,ab. |
| 34. older people.ti,ab. |
| 35. older patient*.ti,ab. |
| 36. older women.ti,ab. |
| 37. older men.ti,ab. |
| 38. older individual*.ti,ab. |
| 39. geriatric*.ti,ab. |
| 40. gerontology.ti,ab. |
| 41. senior citizen.ti,ab. |
| 42. senior citizens.ti,ab. |
| 43. seniors.ti,ab. |
| 44. septuagenarian*.ti,ab. |
| 45. octogenarian*.ti,ab. |
| 46. nonagenarian*.ti,ab. |
| 47. exp aged/ |
| 48. elders.ti,ab. |
| 49. older person*.ti,ab. |
| 50. 29 or 30 or 31 or 32 or 33 or 34 or 35 or 36 or 37 or 38 or 39 or 40 or 41 or 42 or 43 or 44 or 45 or 46 or 47 or 48 or 49 |
| 51. ((developing or less* developed or under developed or underdeveloped or middle income or low* income or underserved or under served or deprived or poor*) adj (economy or economies)).ti,ab. |
| 52. ((developing or less* developed or under developed or underdeveloped or middle income or low* income or underserved or under served or deprived or poor*) adj (countr* or nation? or population? or world)).ti,ab. |
| 53. (low* adj (gdp or gnp or gross domestic or gross national)).ti,ab. |
| 54. (low adj3 middle adj3 countr*).ti,ab. |
| 55. (lmic or lmics or third world or lami countr*).ti,ab. |
| 56. transitional countr*.ti,ab. |
| 57. Cambodia/ |
| 58. (cambodia or khmer republic).mp. |
| 59. North Korea/ |
| 60. (north korea or (democratic people* republic adj2 korea)).mp. |
| 61. Myanmar/ |
| 62. (myanmar or burma).mp. |
| 63. Tajikistan/ |
| 64. (tajikistan or tadzhik or tadzhikistan or tajikistan).mp. |
| 65. Haiti/ |
| 66. Haiti.mp. |
| 67. Afghanistan/ |
| 68. Afghanistan.mp. |
| 69. Bangladesh/ |
| 70. Bangladesh.mp. |
| 71. Nepal/ |
| 72. Nepal.mp. |
| 73. Benin/ |
| 74. (Benin or Dahomey).mp. |
| 75. Burkina Faso/ |
| 76. (Burkina Faso or Burkina Fasso or Upper Volta).mp. |
| 77. Burundi/ |
| 78. Burundi.mp. |
| 79. Central African Republic/ |
| 80. (Central African Republic or Ubangi-Shari).mp. |
| 81. Chad/ |
| 82. Chad.mp. |
| 83. Comoros/ |
| 84. (Comoros or Comoro Islands or Mayotte or Iles Comores).mp. |
| 85. ((democratic republic adj2 congo) or belgian congo or zaire).mp. |
| 86. Eritrea/ |
| 87. Eritrea.mp. |
| 88. Ethiopia/ |
| 89. Ethiopia.mp. |
| 90. Gambia/ |
| 91. Gambia.mp. |
| 92. Guinea/ |
| 93. (Guinea not (New Guinea or Guinea Pig* or Guinea Fowl)).mp. |
| 94. Guinea-Bissau/ |
| 95. (Guinea-Bissau or Portuguese Guinea).mp. |
| 96. Kenya/ |
| 97. Kenya.mp. |
| 98. Liberia/ |
| 99. Liberia.mp. |
| 100. Madagascar/ |
| 101. (Madagascar or Malagasy Republic).mp. |
| 102. Malawi/ |
| 103. (Malawi or Nyasaland).mp. |
| 104. Mali/ |
| 105. Mali.mp. |
| 106. Mozambique/ |
| 107. (Mozambique or Portuguese East Africa).mp. |
| 108. Niger/ |
| 109. (Niger not (Aspergillus or Peptococcus or Schizothorax or Cruciferae or Gobius or Lasius or Agelastes or Melanosuchus or radish or Parastromateus or Orius or Apergillus or Parastromateus or Stomoxys)).mp. |
| 110. Rwanda/ |
| 111. (Rwanda or Ruanda).mp. |
| 112. Sierra Leone/ |
| 113. Sierra Leone.mp. |
| 114. Somalia/ |
| 115. Somalia.mp. |
| 116. Tanzania/ |
| 117. (Tanzania or Zanzibar).mp. |
| 118. Togo/ |
| 119. (Togo or Togolese Republic).mp. |
| 120. Uganda/ |
| 121. Uganda.mp. |
| 122. Zimbabwe/ |
| 123. (Zimbabwe or Rhodesia).mp. |
| 124. Indonesia/ |
| 125. indonesia.mp. |
| 126. kiribati/ |
| 127. Kiribati.mp. |
| 128. "Federated States of Micronesia"/ |
| 129. micronesia.mp. |
| 130. Laos/ |
| 131. (laos or (lao adj2 democratic republic)).mp. |
| 132. marshall islands/ |
| 133. marshall island*.mp. |
| 134. Mongolia/ |
| 135. (mongolia or mongolian people* republic).mp. |
| 136. Papua New Guinea/ |
| 137. Papua New Guinea.mp. |
| 138. Philippines/ |
| 139. Philippines.mp. |
| 140. Samoa/ |
| 141. samoa.mp. |
| 142. solomon islands/ |
| 143. Solomon Islands.mp. |
| 144. Melanesia/ |
| 145. melanesia.mp. |
| 146. (Timor-Leste or East Timor).mp. |
| 147. Vanuatu/ |
| 148. Vanuatu.mp. |
| 149. Viet Nam/ |
| 150. (Viet Nam or Vietnam).mp. |
| 151. Armenia/ |
| 152. Armenia.mp. |
| 153. (georgia not (georgia adj3 state)).mp. |
| 154. "yugoslavia (pre-1992)"/ or yugoslavia/ |
| 155. Kosovo/ |
| 156. kosovo.mp. |
| 157. Kyrgyzstan/ |
| 158. (kyrgyzstan or kyrgyz republic or kirghizia or kirghiz).mp. |
| 159. Moldova/ |
| 160. Moldova.mp. |
| 161. Ukraine/ |
| 162. Ukraine.mp. |
| 163. Uzbekistan/ |
| 164. Uzbekistan.mp. |
| 165. Bolivia/ |
| 166. Bolivia.mp. |
| 167. El Salvador/ |
| 168. salvador.mp. |
| 169. Guatemala/ |
| 170. Guatemala.mp. |
| 171. Guyana/ |
| 172. Guyana.mp. |
| 173. Honduras/ |
| 174. Honduras.mp. |
| 175. Nicaragua/ |
| 176. Nicaragua.mp. |
| 177. Paraguay/ |
| 178. Paraguay.mp. |
| 179. Djibouti/ |
| 180. (Djibouti or French Somaliland).mp. |
| 181. Egypt/ |
| 182. Egypt.mp. |
| 183. Morocco/ |
| 184. Morocco.mp. |
| 185. Syrian Arab Republic/ |
| 186. (Syria or Syrian Arab Republic).mp. |
| 187. Gaza.mp. |
| 188. Yemen/ |
| 189. Yemen.mp. |
| 190. or/179-189 |
| 191. Bhutan/ |
| 192. Bhutan.mp. |
| 193. India/ |
| 194. India.mp. |
| 195. Pakistan/ |
| 196. Pakistan.mp. |
| 197. Sri Lanka/ |
| 198. (Sri Lanka or Ceylon).mp. |
| 199. Cameroon/ |
| 200. Cameroon.mp. |
| 201. Cape Verde/ |
| 202. (Cape Verde or Cabo Verde).mp. |
| 203. Congo/ |
| 204. (congo not ((democratic republic adj3 congo) or congo red or crimean-congo)).mp. |
| 205. Cote d'Ivoire/ |
| 206. (Cote d'Ivoire or Ivory Coast).mp. |
| 207. Ghana/ |
| 208. (Ghana or Gold Coast).mp. |
| 209. Lesotho/ |
| 210. (Lesotho or Basutoland).mp. |
| 211. Mauritania/ |
| 212. Mauritania.mp. |
| 213. Nigeria/ |
| 214. Nigeria.mp. |
| 215. "sao tome and principe"/ |
| 216. (sao tome adj2 principe).mp. |
| 217. Senegal/ |
| 218. Senegal.mp. |
| 219. Sudan/ |
| 220. Sudan.mp. |
| 221. Swaziland/ |
| 222. Swaziland.mp. |
| 223. Zambia/ |
| 224. (Zambia or Northern Rhodesia).mp. |
| 225. American Samoa/ |
| 226. american samoa.mp. |
| 227. China/ |
| 228. (china or (chinese adj3 republic)).mp. |
| 229. Fiji/ |
| 230. fiji.mp. |
| 231. Malaysia/ |
| 232. malaysia.mp. |
| 233. marshall islands/ |
| 234. marshall islands.mp. |
| 235. Palau/ |
| 236. Palau.mp. |
| 237. Thailand/ |
| 238. (Thailand or Siam).mp. |
| 239. tuvalu/ |
| 240. Tuvalu.mp. |
| 241. Albania/ |
| 242. Albania.mp. |
| 243. Azerbaijan/ |
| 244. Azerbaijan.mp. |
| 245. Belarus/ |
| 246. (belarus or byelarus or belorussia).mp. |
| 247. bosnia.mp. |
| 248. Bulgaria/ |
| 249. Bulgaria.mp. |
| 250. Hungary/ |
| 251. hungary.mp. |
| 252. Kazakhstan/ |
| 253. (Kazakhstan or kazakh).mp. |
| 254. Macedonia.mp. |
| 255. Montenegro.mp. |
| 256. Romania/ |
| 257. Romania.mp. |
| 258. Serbia/ |
| 259. serbia.mp. |
| 260. turkey.mp. not "turkey (bird)"/ |
| 261. Turkmenistan/ |
| 262. Turkmenistan.mp. |
| 263. Argentina/ |
| 264. Argentina.mp. |
| 265. Belize/ |
| 266. (Belize or British Honduras).mp. |
| 267. Brazil/ |
| 268. Brazil.mp. |
| 269. Colombia/ |
| 270. Colombia.mp. |
| 271. Costa Rica/ |
| 272. Costa Rica.mp. |
| 273. Cuba/ |
| 274. Cuba.mp. |
| 275. Dominica/ |
| 276. Dominica.mp. |
| 277. Dominican Republic/ |
| 278. Dominican Republic.mp. |
| 279. Ecuador/ |
| 280. Ecuador.mp. |
| 281. Grenada/ |
| 282. Grenada.mp. |
| 283. Jamaica/ |
| 284. Jamaica.mp. |
| 285. Mexico/ |
| 286. Mexico.mp. |
| 287. Panama/ |
| 288. Panama.mp. |
| 289. Peru/ |
| 290. Peru.mp. |
| 291. Saint Lucia/ |
| 292. (St Lucia or Saint Lucia).mp. |
| 293. "Saint Vincent and the Grenadines"/ |
| 294. Grenadines.mp. |
| 295. Suriname/ |
| 296. Suriname.mp. |
| 297. Venezuela/ |
| 298. Venezuela.mp. |
| 299. Algeria/ |
| 300. Algeria.mp. |
| 301. Iran/ |
| 302. Iran.mp. |
| 303. Iraq/ |
| 304. Iraq.mp. |
| 305. Jordan/ |
| 306. Jordan.mp. |
| 307. Lebanon/ |
| 308. Lebanon.mp. |
| 309. Libyan Arab Jamahiriya/ |
| 310. (Libya or libyan arab jamahiriya).mp. |
| 311. Tunisia/ |
| 312. Tunisia.mp. |
| 313. maldives/ |
| 314. Maldives.mp. |
| 315. Angola/ |
| 316. Angola.mp. |
| 317. Botswana/ |
| 318. (Botswana or Bechuanaland or Kalahari).mp. |
| 319. Gabon/ |
| 320. (Gabon or Gabonese Republic).mp. |
| 321. Mauritius/ |
| 322. (Mauritius or Agalega Islands).mp. |
| 323. Namibia/ |
| 324. Namibia.mp. |
| 325. Seychelles/ |
| 326. Seychelles.mp. |
| 327. South Africa/ |
| 328. South Africa.mp. |
| 329. exp Developing Countries/ |
| 330. Congo Democratic Republic.gl. |
| 331. East Timor.gl. |
| 332. exp "Republic of Georgia"/ |
| 333. exp Bosnia-Hercegovina/ |
| 334. exp "Republic of Macedonia"/ |
| 335. exp "Serbia and Montenegro"/ |
| 336. exp Turkey/ |
| 337. 51 or 52 or 53 or 54 or 55 or 56 or 57 or 58 or 59 or 60 or 61 or 62 or 63 or 64 or 65 or 66 or 67 or 68 or 69 or 70 or 71 or 72 or 73 or 74 or 75 or 76 or 77 or 78 or 79 or 80 or 81 or 82 or 83 or 84 or 85 or 86 or 87 or 88 or 89 or 90 or 91 or 92 or 93 or 94 or 95 or 96 or 97 or 98 or 99 or 100 or 101 or 102 or 103 or 104 or 105 or 106 or 107 or 108 or 109 or 110 or 111 or 112 or 113 or 114 or 115 or 116 or 117 or 118 or 119 or 120 or 121 or 122 or 123 or 124 or 125 or 126 or 127 or 128 or 129 or 130 or 131 or 132 or 133 or 134 or 135 or 136 or 137 or 138 or 139 or 140 or 141 or 142 or 143 or 144 or 145 or 146 or 147 or 148 or 149 or 150 or 151 or 152 or 153 or 154 or 155 or 156 or 157 or 158 or 159 or 160 or 161 or 162 or 163 or 164 or 165 or 166 or 167 or 168 or 169 or 170 or 171 or 172 or 173 or 174 or 175 or 176 or 177 or 178 or 179 or 180 or 181 or 182 or 183 or 184 or 185 or 186 or 187 or 188 or 189 or 190 or 191 or 192 or 193 or 194 or 195 or 196 or 197 or 198 or 199 or 200 or 201 or 202 or 203 or 204 or 205 or 206 or 207 or 208 or 209 or 210 or 211 or 212 or 213 or 214 or 215 or 216 or 217 or 218 or 219 or 220 or 221 or 222 or 223 or 224 or 225 or 226 or 227 or 228 or 229 or 230 or 231 or 232 or 233 or 234 or 235 or 236 or 237 or 238 or 239 or 240 or 241 or 242 or 243 or 244 or 245 or 246 or 247 or 248 or 249 or 250 or 251 or 252 or 253 or 254 or 255 or 256 or 257 or 258 or 259 or 260 or 261 or 262 or 263 or 264 or 265 or 266 or 267 or 268 or 269 or 270 or 271 or 272 or 273 or 274 or 275 or 276 or 277 or 278 or 279 or 280 or 281 or 282 or 283 or 284 or 285 or 286 or 287 or 288 or 289 or 290 or 291 or 292 or 293 or 294 or 295 or 296 or 297 or 298 or 299 or 300 or 301 or 302 or 303 or 304 or 305 or 306 or 307 or 308 or 309 or 310 or 311 or 312 or 313 or 314 or 315 or 316 or 317 or 318 or 319 or 320 or 321 or 322 or 323 or 324 or 325 or 326 or 327 or 328 or 329 or 330 or 331 or 332 or 333 or 334 or 335 or 336 |
| 338. 28 and 50 and 337 |

##

## Psych Info (N=177)

| 1. exp Disasters/ |
| --- |
| 2. exp Relief Work/ |
| 3. exp Rescue Work/ |
| 4. exp Emergencies/ |
| 5. exp Disaster Medicine/ |
| 6. exp Mass Casualty Incidents/ |
| 7. exp Emergency Responders/ |
| 8. (humanitarian adj2 (crisis or crises or relief or response or agenc*)).tw. |
| 9. humanitarian.tw. |
| 10. (disaster adj3 (relief or plan$)).tw. |
| 11. ((relief or aid) adj2 work$).tw. |
| 12. Refugees.tw. |
| 13. (refugee or evacuee or evacuated).tw. |
| 14. exp War/ |
| 15. War.tw. |
| 16. ((armed or zone) adj2 conflict$).tw. |
| 17. (conflict affected adj3 (population$ or person$ or communit$)).tw. |
| 18. exp avalanches/ or exp earthquakes/ or exp landslides/ or exp tidal waves/ or exp tsunamis/ or exp volcanic eruptions/ |
| 19. exp cyclonic storms/ or exp droughts/ or exp floods/ or exp tidal waves/ |
| 20. typhoon*.tw. |
| 21. hurricane*.tw. |
| 22. cyclone*.tw. |
| 23. (avalanche$ or earthquake$ or flood or floods or flooding or flooded or landslide$ or tsunami$).tw. |
| 24. (disaster adj2 (natural or victim)).tw. |
| 25. drought$.tw. |
| 26. exp Starvation/ |
| 27. (starvation or famine$).tw. |
| 28. 1 or 2 or 3 or 4 or 5 or 6 or 7 or 8 or 9 or 10 or 11 or 12 or 13 or 14 or 15 or 16 or 17 or 18 or 19 or 20 or 21 or 22 or 23 or 24 or 25 or 26 or 27 |
| 29. exp geriatrics/ |
| 30. elderly.ti,ab. |
| 31. elder.ti,ab. |
| 32. aging.ti,ab. |
| 33. older adult*.ti,ab. |
| 34. older people.ti,ab. |
| 35. older patient*.ti,ab. |
| 36. older women.ti,ab. |
| 37. older men.ti,ab. |
| 38. older individual*.ti,ab. |
| 39. geriatric*.ti,ab. |
| 40. gerontology.ti,ab. |
| 41. senior citizen.ti,ab. |
| 42. senior citizens.ti,ab. |
| 43. seniors.ti,ab. |
| 44. septuagenarian*.ti,ab. |
| 45. octogenarian*.ti,ab. |
| 46. nonagenarian*.ti,ab. |
| 47. exp aged/ |
| 48. elders.ti,ab. |
| 49. older person*.ti,ab. |
| 50. 29 or 30 or 31 or 32 or 33 or 34 or 35 or 36 or 37 or 38 or 39 or 40 or 41 or 42 or 43 or 44 or 45 or 46 or 47 or 48 or 49 |
| 51. ((developing or less* developed or under developed or underdeveloped or middle income or low* income or underserved or under served or deprived or poor*) adj (economy or economies)).ti,ab. |
| 52. ((developing or less* developed or under developed or underdeveloped or middle income or low* income or underserved or under served or deprived or poor*) adj (countr* or nation? or population? or world)).ti,ab. |
| 53. (low* adj (gdp or gnp or gross domestic or gross national)).ti,ab. |
| 54. (low adj3 middle adj3 countr*).ti,ab. |
| 55. (lmic or lmics or third world or lami countr*).ti,ab. |
| 56. transitional countr*.ti,ab. |
| 57. (cambodia or khmer republic).mp. |
| 58. (north korea or (democratic people* republic adj2 korea)).mp. |
| 59. (myanmar or burma).mp. |
| 60. (tajikistan or tadzhik or tadzhikistan or tajikistan).mp. |
| 61. Haiti.mp. |
| 62. Afghanistan.mp. |
| 63. Bangladesh.mp. |
| 64. Nepal.mp. |
| 65. (Benin or Dahomey).mp. |
| 66. (Burkina Faso or Burkina Fasso or Upper Volta).mp. |
| 67. Burundi.mp. |
| 68. (Central African Republic or Ubangi-Shari).mp. |
| 69. Chad.mp. |
| 70. (Comoros or Comoro Islands or Mayotte or Iles Comores).mp. |
| 71. ((democratic republic adj2 congo) or belgian congo or zaire).mp. |
| 72. Eritrea.mp. |
| 73. Ethiopia.mp. |
| 74. Gambia.mp. |
| 75. Guinea/ |
| 76. (Guinea not (New Guinea or Guinea Pig* or Guinea Fowl)).mp. |
| 77. (Guinea-Bissau or Portuguese Guinea).mp. |
| 78. Kenya.mp. |
| 79. Liberia.mp. |
| 80. (Madagascar or Malagasy Republic).mp. |
| 81. (Malawi or Nyasaland).mp. |
| 82. Mali.mp. |
| 83. (Mozambique or Portuguese East Africa).mp. |
| 84. (Niger not (Aspergillus or Peptococcus or Schizothorax or Cruciferae or Gobius or Lasius or Agelastes or Melanosuchus or radish or Parastromateus or Orius or Apergillus or Parastromateus or Stomoxys)).mp. |
| 85. (Rwanda or Ruanda).mp. |
| 86. Sierra Leone.mp. |
| 87. Somalia.mp. |
| 88. (Tanzania or Zanzibar).mp. |
| 89. (Togo or Togolese Republic).mp. |
| 90. Uganda.mp. |
| 91. (Zimbabwe or Rhodesia).mp. |
| 92. indonesia.mp. |
| 93. Kiribati.mp. |
| 94. micronesia.mp. |
| 95. (laos or (lao adj2 democratic republic)).mp. |
| 96. marshall island*.mp. |
| 97. (mongolia or mongolian people* republic).mp. |
| 98. Papua New Guinea.mp. |
| 99. Philippines.mp. |
| 100. samoa.mp. |
| 101. Solomon Islands.mp. |
| 102. melanesia.mp. |
| 103. (Timor-Leste or East Timor).mp. |
| 104. Vanuatu.mp. |
| 105. (Viet Nam or Vietnam).mp. |
| 106. Armenia.mp. |
| 107. (georgia not (georgia adj3 state)).mp. |
| 108. kosovo.mp. |
| 109. (kyrgyzstan or kyrgyz republic or kirghizia or kirghiz).mp. |
| 110. Moldova.mp. |
| 111. Ukraine.mp. |
| 112. Uzbekistan.mp. |
| 113. Bolivia.mp. |
| 114. salvador.mp. |
| 115. Guatemala.mp. |
| 116. Guyana.mp. |
| 117. Honduras.mp. |
| 118. Nicaragua.mp. |
| 119. Paraguay.mp. |
| 120. (Djibouti or French Somaliland).mp. |
| 121. Egypt.mp. |
| 122. Morocco.mp. |
| 123. (Syria or Syrian Arab Republic).mp. |
| 124. Gaza.mp. |
| 125. Yemen.mp. |
| 126. Bhutan.mp. |
| 127. India.mp. |
| 128. Pakistan.mp. |
| 129. (Sri Lanka or Ceylon).mp. |
| 130. Cameroon.mp. |
| 131. (Cape Verde or Cabo Verde).mp. |
| 132. (congo not ((democratic republic adj3 congo) or congo red or crimean-congo)).mp. |
| 133. (Cote d'Ivoire or Ivory Coast).mp. |
| 134. (Ghana or Gold Coast).mp. |
| 135. (Lesotho or Basutoland).mp. |
| 136. Mauritania.mp. |
| 137. Nigeria.mp. |
| 138. (sao tome adj2 principe).mp. |
| 139. Senegal.mp. |
| 140. Sudan.mp. |
| 141. Swaziland.mp. |
| 142. (Zambia or Northern Rhodesia).mp. |
| 143. american samoa.mp. |
| 144. (china or (chinese adj3 republic)).mp. |
| 145. fiji.mp. |
| 146. malaysia.mp. |
| 147. marshall islands.mp. |
| 148. Palau.mp. |
| 149. (Thailand or Siam).mp. |
| 150. Tuvalu.mp. |
| 151. Albania.mp. |
| 152. Azerbaijan.mp. |
| 153. (belarus or byelarus or belorussia).mp. |
| 154. bosnia.mp. |
| 155. Bulgaria.mp. |
| 156. hungary.mp. |
| 157. (Kazakhstan or kazakh).mp. |
| 158. Macedonia.mp. |
| 159. Montenegro.mp. |
| 160. Romania.mp. |
| 161. serbia.mp. |
| 162. turkey.mp. not "turkey (bird)"/ |
| 163. Turkmenistan.mp. |
| 164. Argentina.mp. |
| 165. (Belize or British Honduras).mp. |
| 166. Brazil.mp. |
| 167. Colombia.mp. |
| 168. Costa Rica.mp. |
| 169. Cuba.mp. |
| 170. Dominica.mp. |
| 171. Dominican Republic.mp. |
| 172. Ecuador.mp. |
| 173. Grenada.mp. |
| 174. Jamaica.mp. |
| 175. Mexico.mp. |
| 176. Panama.mp. |
| 177. Peru.mp. |
| 178. (St Lucia or Saint Lucia).mp. |
| 179. Grenadines.mp. |
| 180. Suriname.mp. |
| 181. Venezuela.mp. |
| 182. Algeria.mp. |
| 183. Iran.mp. |
| 184. Iraq.mp. |
| 185. Jordan.mp. |
| 186. Lebanon.mp. |
| 187. (Libya or libyan arab jamahiriya).mp. |
| 188. Tunisia.mp. |
| 189. Maldives.mp. |
| 190. Angola.mp. |
| 191. (Botswana or Bechuanaland or Kalahari).mp. |
| 192. (Gabon or Gabonese Republic).mp. |
| 193. (Mauritius or Agalega Islands).mp. |
| 194. Namibia.mp. |
| 195. Seychelles.mp. |
| 196. South Africa.mp. |
| 197. exp Developing Countries/ |
| 198. 51 or 52 or 53 or 54 or 55 or 56 or 57 or 58 or 59 or 60 or 61 or 62 or 63 or 64 or 65 or 66 or 67 or 68 or 69 or 70 or 71 or 72 or 73 or 74 or 75 or 76 or 77 or 78 or 79 or 80 or 81 or 82 or 83 or 84 or 85 or 86 or 87 or 88 or 89 or 90 or 91 or 92 or 93 or 94 or 95 or 96 or 97 or 98 or 99 or 100 or 101 or 102 or 103 or 104 or 105 or 106 or 107 or 108 or 109 or 110 or 111 or 112 or 113 or 114 or 115 or 116 or 117 or 118 or 119 or 120 or 121 or 122 or 123 or 124 or 125 or 126 or 127 or 128 or 129 or 130 or 131 or 132 or 133 or 134 or 135 or 136 or 137 or 138 or 139 or 140 or 141 or 142 or 143 or 144 or 145 or 146 or 147 or 148 or 149 or 150 or 151 or 152 or 153 or 154 or 155 or 156 or 157 or 158 or 159 or 160 or 161 or 162 or 163 or 164 or 165 or 166 or 167 or 168 or 169 or 170 or 171 or 172 or 173 or 174 or 175 or 176 or 177 or 178 or 179 or 180 or 181 or 182 or 183 or 184 or 185 or 186 or 187 or 188 or 189 or 190 or 191 or 192 or 193 or 194 or 195 or 196 or 197 |
| 199. 28 and 50 and 198 |

## IBSS (N=1895)

| ((all("older patient*") AND peer(yes)) OR (all("older women") AND peer(yes)) OR (all("older men") AND peer(yes)) OR (all("older individual*") AND peer(yes)) OR (SU.EXACT.EXPLODE("Geriatrics") AND peer(yes)) OR ((SU.EXACT.EXPLODE("Aged") OR SU.EXACT.EXPLODE("Care of the aged")) AND peer(yes)) OR (all(elderly) AND peer(yes)) OR (all(elder) AND peer(yes)) OR (all(aging) AND peer(yes)) OR (all("older adult*") AND peer(yes)) OR (all("older people") AND peer(yes)) OR (all("geriatric*") AND peer(yes)) OR (all("gerontology") AND peer(yes)) OR (all(" senior citizen") AND peer(yes)) OR (all(" senior citizens") AND peer(yes)) OR (all("seniors") AND peer(yes)) OR (all(" septuagenarian*") AND peer(yes)) OR (all("octogenarian*") AND peer(yes)) OR (all("nonagenarian*") AND peer(yes)) OR (all(elders) AND peer(yes)) OR (all("older person*") AND peer(yes))) AND (((SU.EXACT.EXPLODE("Disasters") OR SU.EXACT.EXPLODE("Natural disasters") OR SU.EXACT.EXPLODE("Disaster relief")) AND peer(yes)) OR (all((humanitarian NEAR/3 (crisis OR crises OR relief OR response OR agenc*))) AND peer(yes)) OR (all((humanitarian NEAR/3 (crisis OR crises OR relief OR response OR agency*))) AND peer(yes)) OR (all(humanitarian) AND peer(yes)) OR (all((disaster NEAR/3 (relief OR plan*))) AND peer(yes)) OR (all(((relief OR aid) NEAR/2 work*)) AND peer(yes)) OR (all(Refugees) AND peer(yes)) OR (all((refugee OR evacuee OR evacuated)) AND peer(yes)) OR (all(war) AND peer(yes)) OR ((SU.exact("WAR") OR ORG.exact("WAR")) AND (SU.exact("CONFLICTS") OR SU.exact("CONFLICT"))) OR (all(((armed OR zone) NEAR/2 conflict*)) AND peer(yes)) OR (all(("conflict affected" NEAR/3 (population* OR person* OR communit*))) AND peer(yes)) OR (all(typhoon*) AND peer(yes)) OR (all(hurricane*) AND peer(yes)) OR (all(cyclone*) AND peer(yes)) OR (all((avalanche* OR earthquake* OR flood OR floods OR flooding OR flooded OR landslide* OR tsunami*)) AND peer(yes)) OR (all((disaster NEAR/2 (natural OR victim))) AND peer(yes)) OR (all(drought*) AND peer(yes)) OR (all((starvation OR famine*)) AND peer(yes))) |
| --- |
