# Supplementary material for: Health needs of older populations affected by humanitarian crises in low- and middle-income countries: a systematic review
Source: Confl Health. 2017 Dec 11;11:29. doi: 10.1186/s13031-017-0133-x (PMC5724338; doi:10.1186/s13031-017-0133-x)
Supplement: Supplementary file 3 — Detailed Quality Appraisal Results. (DOCX 23 kb) [file 13031_2017_133_MOESM3_ESM.docx]

**Additional file 3: Detailed Quality Appraisal Results**

### Table A: Newcastle - Ottawa Quality Assessment Scale for cross-sectional studies

|  | Representativeness of sample (maximum 1 star) | Sample size  (maximum 1 star) | Non-respondents  (maximum 1 star) | Ascertainment of the  exposure (maximum 2 stars) | Confounding factors controlled (maximum 2 stars) | Assessment of the outcome (maximum 2 stars) | Statistical test  (maximum 1 star) | Total score (number of stars) |
| --- | --- | --- | --- | --- | --- | --- | --- | --- |
| Andre et al, 2013 | - | - | - | * | - | ** | - | 3 |
| Ardalan et al, 2011 (a) | * | - | * | ** | * | * | * | 7 |
| Ardalen et al, 2011 (b) | * | - | * | * | * | ** | * | 7 |
| Arlappa et al, 2009 | * | - | - | * | - | ** | - | 4 |
| Cao, X. 2014 | - | * | - | ** | - | * | * | 5 |
| Chaaya et al, 2007 | * | - | * | * | ** | * | * | 7 |
| Chan et al. 2009(a) | * | - | - | * | - | ** | - | 4 |
| Chan, 2009(b) | * | - | - | ** | - | * | - | 4 |
| Chen et al 2012 | * | * | * | * | ** | * | * | 8 |
| Godfrey & Kalache, 1989 | * | - | - | * | - | * | - | 3 |
| Goenjian et al, 1994 | - | - | - | ** | - | ** | - | 4 |
| Handicap International & HelpAge International, 2014 | * | * | - | * | - | - | * | 4 |
| Havelka et al, 1995 | * | - | - | * | - | * | - | 3 |
| Jia et al. 2010 | * | * | * | ** | * | * | * | 8 |
| Johns Hopkins & Institute for Policy Studies, 2012 | * | * | * | ** | - | * | - | 6 |
| Kohn et al, 2005 | * | - | - | ** | ** | * | * | 7 |
| Li et al. 2011 | * | * | * | ** | ** | ** | * | 10 |
| Lutala et al, 2010 | * | * | - | - | - | * | - | 3 |
| Nomura et al. 2010 | * | - | - | ** | ** | * | * | 7 |
| Pieterse et al, 1998 | * | - | * | * | * | ** | - | 6 |
| Pieterse et al, 2002 | - | - | * | ** | ** | ** | * | 8 |
| Pieterse & Ismail, 2003 | * | - | * | * | - | * | - | 4 |
| Prueksaritanond, S et al (2007) |  |  |  |  |  | * | * | 2 |
| Ramji & Thoner, 1991 | - | - | - | - | - | ** | - | 2 |
| Strong et al. 2015 | - | * | * | * | - | * | * | 5 |
| Viswanath et al 2012 | - | * | * | * | - | ** | - | 5 |
| Wen et al. 2010 | * | * | - | * | - | ** | * | 6 |
| Wu et al. 2015 | * | * | * | - | ** | ** | * | 8 |
| Zhang et al. 2012. (a) | * | * | - | * | ** | * | * | 7 |
| Zhang et al. 2012 (b) | * | * | * | ** | ** | * | * | 9 |
| Zhang et al. 2012. (c) |  |  |  | ** | * | * | * | 5 |
| *Quality assessment per NOS criteria for cross-sectional studies. Stars (*) awarded if study reached threshold of high quality for that category. Letters and descriptions given to cross-reference with NOS coding manual. For further information on NOS scoring for cross-sectional studies see* [*main*](#_ENREF_16) *manuscript.* | | | | | | | | |

#### **Table B: Newcastle - Ottawa Quality Assessment Scale for cohort studies**

|  | Representativeness of exposed (maximum 1 star) | Selection of non-exposed  (maximum 1 star) | Ascertainment of exposure  (maximum 1 star) | Outcome not present at start  (maximum 1 star) | Confounding factors controlled?  (maximum 2 stars) | Assessment of outcome  (maximum 1 star) | Follow up long enough?  (maximum 1 star) | Adequacy of follow up  (maximum 1 star) | Total |
| --- | --- | --- | --- | --- | --- | --- | --- | --- | --- |
| Sibai et al. 2007 | * | * | * | * | ** | - | * | * | 8 |
| Sibai et al. 2001 | * | * | * | * | ** | * | * | * | 9 |
| Wong et al, 2015 |  |  | * | * |  | * | * | * | 5 |
| *Quality assessment per NOS criteria for cohort studies. Stars (*) awarded if study reached threshold of high quality for that category. For further information on the scoring scale see main manuscript* | | | | | | | | | |

**Table C: CASP - Critical Appraisal of Qualitative research**

|  | 1. Clear statement of aims? | 2. Qualitative methodology appropriate? | 3. Research design appropriate to address aims? | 4. Recruitment strategy appropriate to the aims? | 5. Data collected in a way that addressed research issue? | 6. Relationship between researcher and participants adequately considered? | 7. Ethical issues taken into consideration? | 8. Data analysis sufficiently rigorous? | 9. Clear statement of findings? | 10. How valuable is the research? | TOTAL |
| --- | --- | --- | --- | --- | --- | --- | --- | --- | --- | --- | --- |
| Ardalen et al, 2010. | Yes | Yes | Yes | Yes | Yes | No | Yes | Yes | Yes | Good | 9 |
| Duggan et al. 2010. | Yes | Yes | Yes | No | Yes | No | Yes | Yes | Yes | Somewhat | 7.5 |
| Pieterse & Ismail, 2003. | Yes | Yes | Yes | No | No | No | Yes | No | No | Poor | 4 |
| Johns Hopkins & Institute for Policy Studies, 2012 | Yes | Yes | Yes | Yes | Yes | No | No | Yes | Yes | Good | 8 |
